# Reversible *trans*-to-*cis* photoisomerization and irreversible photocyclization reactions of a Co-coordinated stilbene derivative on chiral di-β-diketonate lanthanide complexes†

**DOI:** 10.1039/d2ra07133a

**Published:** 2023-01-13

**Authors:** Ziting Hou, Yanji Huang, Yushan Ruan, Han Xu, Yu Tan, Li-Rong Lin, Zhen-yi Wu

**Affiliations:** a Department of Chemistry, College of Chemistry and Chemical Engineering, Xiamen University Xiamen 361005 P. R. China linlr@xmu.edu.cn; b School of Pharmaceutical Science & Yunnan Key Laboratory of Pharmacology for Natural Products, Kunming Medical University Kunming 650500 P. R. China

## Abstract

Six lanthanide complexes constructed from two chiral β-diketonates (d/l-fbc = 3-heptafluorobutyry*l-*(+)/(−)-camphorate), the stilbene derivative (*E*)-*N*′,*N*′-bis(pyridin-2-ylmethyl)-4-styrylbenzoyl hydrazide (L), a trifluoroacetate anion (CF_3_CO_2_^−^), and one water molecule, namely [Ln(d/l-fbc)_2_(L)(CF_3_CO_2_)]·H_2_O (LnC_57_H_54_F_17_N_4_O_8_, Ln = La (1, d-fbc), La (2, l*-*fbc), Sm (3, d-fbc), Eu (4, d-fbc), Eu (5, l*-*fbc), and Tb (6, d-fbc), were synthesized and characterized by single-crystal X-ray diffraction, ^1^H-NMR, elemental analysis, IR and UV-vis spectroscopy, and thermal gravimetric analysis. The photoisomerization reactions of these complexes were systematically studied by means of experimental and theoretical calculations. Crystals of complexes 1, 2, 3, and 4 were obtained and belong to the monoclinic crystal system and the *C*2 chiral space group. The Λ- and Δ-diastereomers coexist in their crystals and no apparent bisignate couplets are observed in their ECD spectra. Among the complexes, the photocyclization reaction is followed by the *trans*-to-*cis* photoisomerization reaction and competes with the *trans*-to-*cis* photoisomerization, then the photocyclization reaction continues. The photocyclization reaction is irreversible in this stilbene derivative and is delayed in the lanthanide complexes. These results provide a viable strategy for the design of promising new stilbene-attached dual-functional lanthanide-based optical-switching materials.

## Abstract 2

Six lanthanide complexes constructed from β-diketonates, a stilbene derivative, a trifluoroacetate anion and a water molecule, were synthesised and characterised. Their chirality, reversible photoisomerisation and irreversible photocyclisation reactions were also studied.

## Graphical abstract

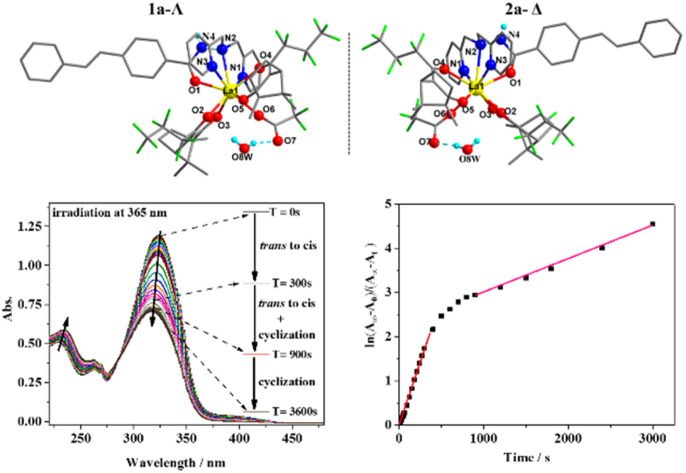

## Introduction

Lanthanide complexes have a wide range of applications in analytical chemistry, catalysis, medicine, and materials.^1–21^ The development of lanthanide complexes with multi-output properties by external stimulation, especially with reversible output, is of great significance in the field of smart materials.^22–26^ Of the various external stimuli, such light energy, electrical energy, and chemical energy, light is a clean energy stimulus source that has the characteristics of independent changeable modality, precise control, low energy, environmental benignity, and remote control. Light is the most suitable and convenient stimuli and can activate the specified molecular structure in a short time.^27–31^ Photochromic molecules such as spiropyrans (SPP) and diarylethenes (DTE) have been introduced into lanthanide complexes to study their photo-stimuli properties, where photo-control of the isomerization of the SPP unit allows reversible absorption/luminescence modulation ability by regulating the fluorescence resonance energy-transfer mechanism between the SPP acceptor and the lanthanide donor.^32–34^ Light-controlled luminescence ON–OFF switchable hybrid materials can be synthesized by loading Eu^3+^ complexes and DTE into monodisperse mesoporous silica nanospheres,^35^ and efficient photo-modulation of Eu(iii) and Yb(iii) luminescences using DTE ligands has been achieved for optical encryption.^36^

**Scheme 1:**
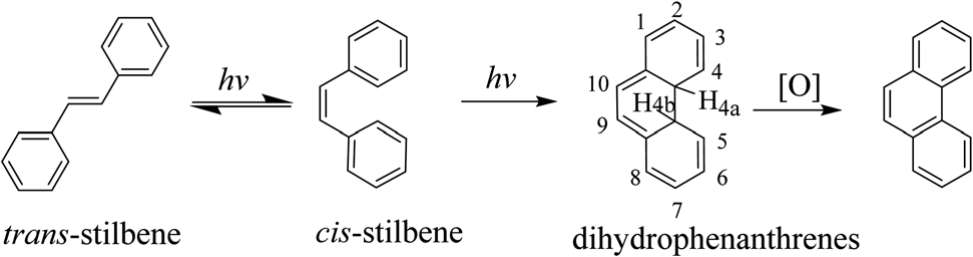
Photochemical transformation of stilbenes.

We have studied a series of lanthanide complexes functionalized with the photoisomerizable azobenzene (AZO) molecule that allow excellent reversible *trans*-to-*cis* photoisomerization of AZO using different-wavelength-light stimuli with good fatigue.^37–39^ However, AZO-functionalized lanthanide complexes exhibit photoisomerization but not switchable luminescence emission, so they are difficult to apply to anti-counterfeiting technology and bioimaging. Stilbene is a model compound for photo-induced *trans*-to-*cis* isomerization, and its photochemistry and photophysics have been extensively studied.^40–44^ Because the *cis* isomer of *trans*-stilbene photoisomerization undergoes photocyclization (Scheme 1), the use of stilbene as a pure *cis*–*trans* isomerization molecular switch has not been as widely researched as that of azobenzene compounds. Since the activation energy (*E*_a_) for the thermal reversion of *cis*-to-*trans* isomerization of stilbene is ∼1.9 eV, thermal reversion of stilbene to the *trans* isomer is practically difficult by light irradiation. Photoreverting isomerization can stabilize its *cis* isomer, and this property has application potential for advanced technological applications in molecular electronics and bioimaging.^45,46^ Therefore, we have focused on designing stilbene derivatives for introduction to lanthanide β-diketonate complexes to obtain regulated luminescence by photo-inducing the structure change of the stilbene group.

**Scheme 2:**
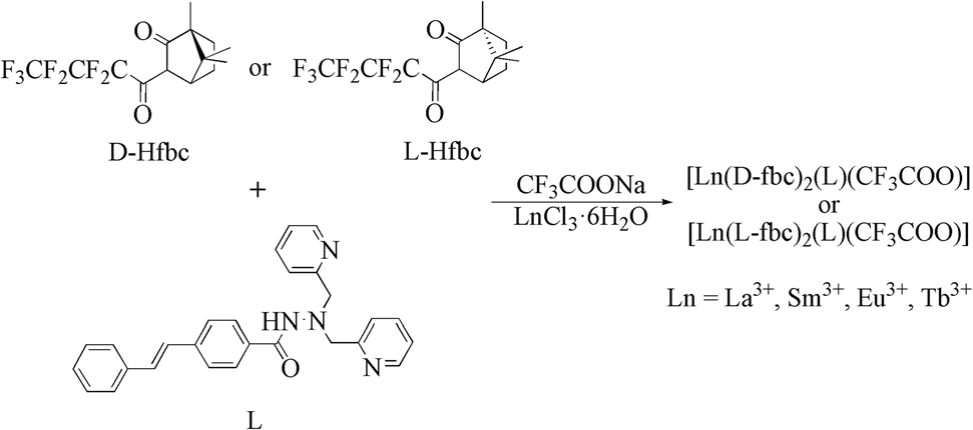
Schematic synthesis procedure for the title complexes.

The as-designed stilbene derivative ligand, *N*′,*N*′-bis(pyridin-2-ylmethyl)-4-styrylbenzoyl hydrazide (L), is a tetradentate chelating ligand that strongly coordinates with lanthanide ions. L was combined with benzoyltrifluoroacetone (Htfd) or 2-thenoyl trifluoroacetone (Htta) for co-coordination with lanthanide ions to construct light-stimuli smart complexes.^47,48^ In our previous studies, it was found that in the stilbene functional group in the lanthanide β-diketonate complexes, its optical properties are influenced by the f-electron transition of the lanthanide ions. Upon coordination with a lanthanide ion, the electron transition of the stilbene ligand is disturbed, thereby affecting its photochemical reactivity and energy transfer. The energy transfer between stilbene ligands and β-diketone ligands and lanthanide ions can regulate the photochemical reaction of the stilbene ligands and the excited-state lifetime of the lanthanide complexes. It has been shown that the luminescence intensity of europium complexes can be reversibly changed from weak to strong by photoisomerization. However, the complexes can undergo photocyclization reactions, which were not confirmed in previous studies.

In order to further understand the *trans*-to-*cis* photoisomerization of stilbene-functionalized lanthanide β-diketonate complexes, especially for the purpose of investigating the effect of chirality on the stilbene functional β-diketonate complexes, we studied the chiral β-diketone molecule 3-(perfluorobutyryl)-camphor in combination with the stilbene derivative ligand L to construct chiral lanthanide (Ln = La^3+^, Sm^3+^, Eu^3+^, Tb^3+^) complexes. We observed that the photocyclization reaction is followed by *trans*-to-*cis* photoisomerization. Detailed knowledge on the effect of chirality on the *trans*-to-*cis* photoisomerization and luminescence properties of the complexes functionalized with stilbene is crucial. These research results provide important theoretical guidance and an experimental basis for the design and synthesis of stilbene-based light-stimuli materials for application to information storage and encryption, molecular switches, anti-counterfeiting, and bio-imprinting.

## Experimental section

### Materials and methods

LnCl_3_·6H_2_O (Ln = La, Sm, Eu and Tb; Heowns, 99.99%), 2-(chloromethyl) pyridine hydrochloride (Adamas Reagent Co., Ltd.), Ethyl stilbene-4-carboxylate (TCI, Shanghai Development Co., Ltd.), 3-(heptafluorobutyryl)-(+)-camphor and 3-(heptafluorobutyryl)-(−)-camphor (d/l-Hfbc, Sigma-Aldrich), acetonitrile-*d*_3_ (Energy Chemical) were used, and all other chemicals were purchased from Sinopharm Chemicals Group Co. The melting point was recorded on an X-4 micromelting point apparatus (Shanghai Precision Scientific Instrument Co. Ltd.) without correction. ESI-MS in methanol solutions was collected on a SHIMADZU LCMS2020 liquid chromatography–MS spectrometer. Elemental analysis was collected on a Vario EL III elemental analyzer. TGA data was collected using an NETZSCH STA 449F5 thermogravimetric analyzer with a heating rate of 10 °C min^−1^ in a temperature range from 30 to 800 °C under a N_2_ atmosphere. The ^1^H NMR spectra were performed on a Bruker Advance II 400 MHz spectrometer in acetonitrile-*d*_3_. Chemical shifts were reported in ppm with TMS signal at 0.0 ppm as the internal reference. FTIR spectra were collected by reflection mode using a Nicolet iS50 spectrometer. The UV-vis spectra were obtained from a Varian Cary 5000 UV-vis-NIR spectrophotometer. ECD spectra were recorded using a Jasco (Easton, MD) J815 spectrometer. The photoisomerization measurements were in the same procedure according to the published methods. Luminescence spectra were scanned using a Hitachi F7000 fluorescence spectrophotometer at room temperature with excitation and emission slits set at 5.0 and 5.0 nm, respectively. Photoirradiation experiments were performed in acetonitrile solution using a Shanghai Yihui ZF-2 UV analyzer. The light intensity was measured using an UV-A radiation meter (Optical Electrical Apparatus Factory, Beijing Normal University). The photoisomerization measurements were performed at a length of 10 mm quartz cell. The concentration of all ligands and complexes used for measurement was 2.0 × 10^−5^ mol L^−1^. The photoisomerization quantum yield (*φ*_iso_) and *trans*-to-*cis* first-order kinetics isomerization rate constant (*k*_iso_) were calculated using previously reported methods.^37^ The data errors for quantum yields and rate constants were within 10%.

### Syntheses of the Lanthanide(III) complexes 1–6 (Scheme 2)

L was prepared in the same procedure according to the published method.^47,48^ The lanthanide complexes were synthesized as follows: 0.21 g of ligand L (0.5 mmol), 0.35 g of d-Hfbc or l*-*Hfbc (0.5 mmol) and 0.07 g sodium trifluoroacetate (CF_3_COONa, 0.5 mmol) were mixed and dissolved in 20 mL of tetrahydrofuran. After heating to 65 °C, 0.4 mol L^−1^ NaOH solution was added dropwise slowly to the solution to adjust the pH to about 7. After that, 0.5 mmol LnCl_3_·6H_2_O in 5 mL of methanol solution was added and refluxing for 10 h under stirring. After the reaction was completed, the resulting solution was concentrated under a reduced pressure to obtain the solids. Then added 20 mL of methanol, filtered the solid, and extracted the filtrate three times with *n*-hexane. The methanol phase was rotated to obtain the pure lanthanide complexes. Crystals were obtained by slow evaporation from absolute ethanol in about 1 month.

#### [La(d-fbc)_2_(L)(CF_3_COO)]·H_2_O (1)

Light yellow solid, yield: 27% (0.18 g); ESI(+)-MS: *m*/*z* (CH_3_OH) = 1253.35 [M^+^](CF_3_COO^−^ was lost), 1019.13 [M^+^](fbc^−^ was lost); ^1^HNMR(400 MHz, Acetonitrile-*d*_3_): *δ* (ppm) 9.76 (s, 1H), 9.43–9.32 (m, 2H), 7.81 (ddq, *J* = 5.9, 3.7, 1.8 Hz, 2H), 7.56–7.49 (m, 2H), 7.43–7.33 (m, 11H), 7.22 (d, *J* = 16.4 Hz, 1H), 7.09 (d, *J* = 16.5 Hz, 1H), 5.06 (dd, *J* = 14.5, 4.0 Hz, 2H), 4.56 (dd, *J* = 14.5, 3.3 Hz, 2H), 2.78 (d, *J* = 4.3 Hz, 2H), 1.61 (ddd, *J* = 13.7, 10.6, 3.5 Hz, 2H), 1.33–1.15 (m, 9H), 0.93 (s, 6H), 0.85 (s, 6H), 0.66 (s, 6H); FT-IR (cm^−1^): 2964 (C–H, m), 1652 (C=O, s), 1627 (C=C, s), 1527 (N–H, s), 1340 (C–N, s), 1210 (C–F, m), 763 (Ph–H, m); elemental analysis calculated for LaC_57_H_54_F_17_N_4_O_8_ (%): C, 49.43; N, 4.05; H, 3.93; found (%): C, 49.55; N, 3.96; H, 3.98.

#### [La(l*-*fbc)_2_(L)(CF_3_COO)]·H_2_O (2)

Light yellow solid, yield: 31% (0.21 g); ESI(+)-MS: *m*/*z* (CH_3_OH) = 1253.35 [M^+^](CF_3_COO^−^ was lost), 1019.13 [M^+^](fbc^−^ was lost); ^1^HNMR (400 MHz, Acetonitrile-*d*_3_) *δ* (ppm) 9.77 (s, 1H), 9.41–9.38 (m, 2H), 7.80 (ddq, *J* = 5.9, 3.7, 1.8 Hz, 2H), 7.54–7.51 (m, 2H), 7.41–7.33 (m, 11H), 7.24 (d, *J* = 16.4 Hz, 1H), 7.11 (d, *J* = 16.5 Hz, 1H), 5.08 (dd, *J* = 14.5, 4.0 Hz, 2H), 4.59 (dd, *J* = 14.5, 3.3 Hz, 2H), 2.77 (d, *J* = 4.3 Hz, 2H), 1.61 (ddd, *J* = 13.7, 10.6, 3.5 Hz, 2H), 1.33–1.15 (m, 9H), 0.93 (s, 6H), 0.85 (s, 6H), 0.66 (s, 6H); FT-IR (cm^−1^): 2964 (C–H, m), 1652 (C=O, s), 1626 (C=C, s), 1527 (N–H, s), 1341 (C–N, s), 1211 (C–F, m), 763 (Ph–H, m); elemental analysis calculated for LaC_57_H_54_F_17_N_4_O_8_ (%): C, 49.43; N, 4.05; H, 3.93; found (%): C, 49.45; N, 3.94; H, 3.93.

#### [Sm(d-fbc)_2_(L)(CF_3_COO)]·H_2_O (3)

Light yellow solid, yield: 25% (0.17 g); ESI(+)-MS: *m*/*z* (CH_3_OH) = 1266.40 [M^+^](CF_3_COO^−^ was lost), 1032.15 [M^+^](fbc^−^ was lost); FT-IR (cm^−1^): 2962 (C–H, m), 1651 (C=O, s), 1605 (C=C, s), 1534 (N–H, s), 1342 (C–N, s), 1210 (C–F, m), 748 (Ph–H, m); elemental analysis calculated for SmC_57_H_54_F_17_N_4_O_8_ (%): C, 49.03; N, 4.01; H, 3.90; found (%): C, 49.12; N, 3.97; H, 3.71.

#### [Eu(d-fbc)_2_(L)(CF_3_COO)]·H_2_O (4)

Light yellow solid, yield: 28% (0.19 g); ESI(+)-MS: *m*/*z* (CH_3_OH) = 1267.35 [M^+^](CF_3_COO^−^ was lost), 1033.20[M^+^](fbc^−^ was lost); FT-IR (cm^−1^): 2960 (C–H, m), 1652 (C=O, s), 1605 (C=C, s), 1533 (N–H, s), 1341 (C–N, s), 1210 (C–F, m), 748 (Ph–H, m); elemental analysis calculated for EuC_57_H_54_F_17_N_4_O_8_ (%): C, 48.97; N, 4.01; H, 3.89; found (%): C, 49.34; N, 4.06; H, 3.96.

#### [Eu(l*-*fbc)_2_(L)(CF_3_COO)]·H_2_O (5)

Light yellow solid, yield: 25% (0.17 g); ESI(+)-MS: *m*/*z* (CH_3_OH) = 1267.35 [M^+^](CF_3_COO^−^ was lost), 1033.20[M^+^](fbc^−^ was lost); FT-IR (cm^−1^):2961 (C–H, m), 1652 (C=O, s), 1606 (C=C, s), 1533 (N–H, s), 1341 (C–N, s), 1211 (C–F, m), 748 (Ph–H, m); elemental analysis calculated for EuC_57_H_54_F_17_N_4_O_8_ (%): C, 48.97; N, 4.01; H, 3.89; found (%): C, 49.24; N, 4.14; H, 3.94.

#### [Tb(d-fbc)_2_(L)(CF_3_COO)]·H_2_O (6)

Light yellow solid, yield: 31% (0.21 g); ESI(+)-MS: *m*/*z* (CH_3_OH) = 1273.40[M^+^](CF_3_COO^−^ was lost), 1039.25[M^+^](fbc^−^ was lost); FT-IR (cm^−1^):2959 (C–H, m), 1654 (C=O, s), 1606 (C=C, s), 1534 (N–H, s), 1342 (C–N, s), 1212 (C–F, m), 748 (Ph–H, m); elemental analysis calculated for TbC_57_H_54_F_17_N_4_O_8_ (%): C, 48.73; N, 3.99; H, 3.87; found (%): C, 48.24; N, 3.96; H, 3.58.

### Structural determination of complexes 1–4 by X-ray diffraction

Single-crystal X-ray diffraction data of complexes 1–4 were collected by a Rigaku Oxford Diffraction XtaLAB Synergy diffractometer with micro-focus sealed X-ray Cu Kα radiation (*λ* = 1.54184 Å) at 100 K in a nitrogen flow cooled with liquid nitrogen. The data reduction and absorption correction were performed with the CrysAlisPro software. The structures were solved by intrinsic phasing (SHELXT) and refined by full-matrix least-squares calculations based on *F*^2^ using the SHELXTL on OLEX2.^49–51^ All non-hydrogen atoms were refined anisotropically.

### Theoretical calculations

The theoretical calculations were performed with the Gaussian 16 program package. Ground-state structures of the ligands and complexes in the acetonitrile solvent were optimized by density functional theory (DFT) calculations using Becke-lee-Yang-Parr (B3LYP) hybrid exchange-correlation functional method.^52^ The acetonitrile solvent was simulated by an implicit solvent model (SMD). The 6-31G(d) basis set was employed for all atoms with the exception of lanthanide ions, for which the relativistic effective core potentials (ECP) and default MWB28 basis sets were used for SDD (Stuttgart Dresden triple zeta ECPs).^53–55^ To verify that the optimized structure was at the energy minimum, frequency calculations were performed. Then the excitations energies and electronic absorption spectra in acetonitrile solvent were computed by time dependent DFT for ligands and complexes. Molecular orbitals were analyzed by a free GaussSum program.^56^

## Results and discussion

### X-ray crystallographic analysis

Suitable crystals of complexes 1–4 with one hydrogen-bonding water in the unit cell were obtained from their ethanol solutions by slow evaporation over one month. Crystal analysis revealed that all four complexes belong to the monoclinic crystal system and the *C*2 chiral space group with flack parameters close to zero, which demonstrates that the determination of absolute configuration is correct. The asymmetric unit within the unit cell consists of two independent molecules. In each independent molecule, the central lanthanide ion of the four complexes is bonded to three nitrogens and one oxygen from the stilbene ligand L, four oxygens from the two depronated Hfbc ligands, and one oxygen from the trifluoroacetate ion to form a stable nine-coordinate structure. For ligand L, the three nitrogen atoms involved in coordination to the lanthanide ion are derived from its two pyridines and hydrazine, and the oxygen atom involved in the coordination is derived from its acyl group, forming tetradentate chelation. The coordination polyhedrons of the four complexes are both distorted tricapped triangular prisms.

**Fig. 1:**
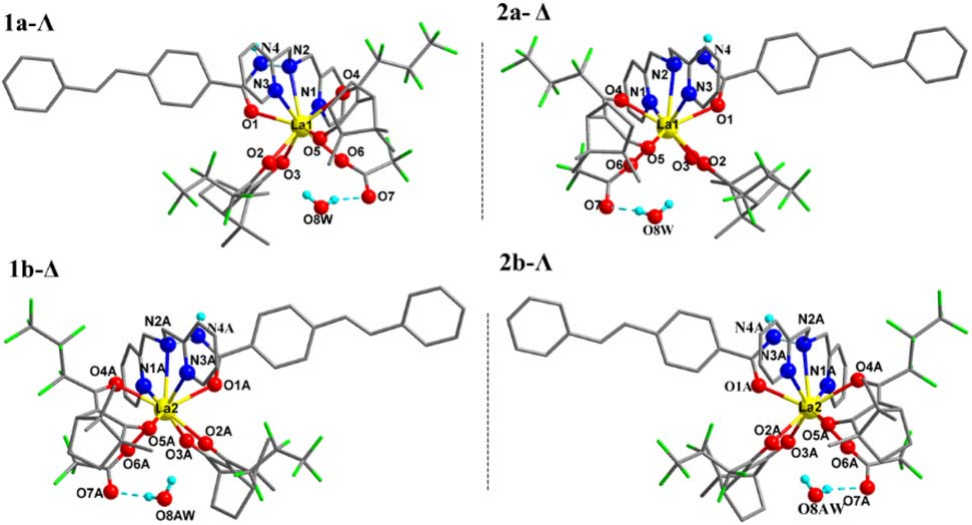
Crystal structures of complexes 1 (1a, 1b, left), 2 (2a, 2b, right) (only hydrogen atoms attached at O8(AW) and N4(A) are shown for clarity).

Table 1 lists the crystallographic parameters of complexes 1–4. Fig. 1 shows the crystal structure of the two independent molecules in the unit cell of complexes 1 and 2, and the polyhedron geometry of the center La(iii) is shown in Fig. S1 in the ESI.† Taking complex 1 as an example, in the asymmetric unit the two independent molecules labeled as 1a and 1b, the left-handed helical arrangement of the two fbc^−^ and L ligands around the La demonstrates that the absolute configuration of La1 is Λ, and the metal-centered chirality of La2 is Δ. It should be pointed that the molecules 1a and 1b with the same chiral ligand d-fbc^−^ are not enantiomers of each other. Complex 2 was prepared using l*-*Hfbc instead of d-Hfbc. Like the previous case, diastereomeric Δ-2 (2a) and Λ-2 (2b) coexist in the crystals. Evidently, 1a and 2a and 1b and 2b are two pairs of enantiomers. Selected bond lengths and bond angles for the four complexes are shown in Tables S1–S4.† The Ln–N bond distances in the four complexes are within the range 2.681–2.853 Å, while the Ln–O bond distances were 2.343–2.549 Å, similar to those of other lanthanide β-diketonate complexes reported previously.^47,48^ In addition, in the unit cell, one hydrogen of a water molecule forms an intermolecular hydrogen bond with the carbonyl oxygen of the trifluoroacetate, and the oxygen of the water forms a hydrogen bonding with the hydrogen of the hydrazide in another molecule to form a stable one-dimensional network. The hydrogen bonding parameters are shown in Table S5.† The crystal structure of the two independent molecules of complexes 3 and 4 and the polyhedron geometry of the central Ln(III) are shown in Fig. S2.†

**Table 1:** Crystallographic data for complexes 1–4

Crystal data	1	2	3	4
Empirical formula	LaC_57_H_54_F_17_N_4_O_8_	LaC_57_H_54_F_17_N_4_O_8_	SmC_57_H_54_F_17_N_4_O_8_	EuC_57_H_54_F_17_N_4_O_8_
Formula weight	1384.95	1384.95	1396.39	1398.00
Crystal system	Monoclinic	Monoclinic	Monoclinic	Monoclinic
Space group	*C*2	*C*2	*C*2	*C*2
*a* (Å)	29.774(2)	29.748(6)	29.487(4)	29.461(2)
*b* (Å)	20.129(10)	20.146(5)	20.097(3)	20.063(10)
*c* (Å)	20.669(10)	20.628(4)	20.698(3)	20.696(10)
*α* (°)	90	90	90	90
*β* (°)	108.433(10)	108.310(2)	108.271(10)	108.2510(10)
*γ* (°)	90	90	90	90
*V* (Å)	11 753.0(13)	11 737.0(5)	11 647.5(3)	11 618.2(13)
*Z*	8	8	8	8
Cal den. (g cm^−3^)	1.565	1.568	1.593	1.598
Abs coe. (mm^−1^)	6.606	6.615	8.568	8.728
*F*(000)	5584.0	5584.0	5624.0	5632.0
2*θ* range (°)	4.51 to 150.65	5.39 to 150.89	4.49 to 150.99	4.49 to 150.62
Refl. col.	72 325	41 953	39 113	77 294
Data/res./para.	22 929/921/1835	18 389/2477/1823	18 886/1909/1926	22 961/1263/1906
GOF	1.024	1.080	1.083	1.054
Final *R*	*R*_1_ = 0.0277	*R*_1_ = 0.0543	*R*_1_ = 0.0514	*R*_1_ = 0.0429
*R* (all data)	*R*_1_ = 0.0281 w*R*_2_ = 0.0709	*R*_1_ = 0.0608, w*R*_2_ = 0.1421	*R*_1_ = 0.0601, w*R*_2_ = 0.1441	*R*_1_ = 0.0453, w*R*_2_ = 0.1153
Flack parameter	−0.0074(13)	−0.003(3)	−0.013(3)	−0.0046(16)

### Thermal analysis of the complexes

We performed thermogravimetric analysis (TGA) on the d-fbc^−^ configuration complexes (1, 3, 4, 6) and the data are listed in Fig. S3.† In the temperature range 240–325 °C, decomposition occurs with mass losses of 34.18% for complex 1, 30.45% for complex 3, 35.41% for complex 4, and 30.80% for complex 6. The percentages are similar to the theoretical calculated values for the loss of one trifluoroacetate and one d-fbc^−^ (33.51% for 1, 33.34% for 3, 33.31% for 4, and 33.18% for 6). The TGA curves of the five complexes are roughly the same, and the difference between the actual value of mass loss and the theoretical value of mass loss may be related to the measurement error of the instrument and the differences in the stabilities of the complexes. All complexes further decompose continuously after 325 °C to 800 °C. TGA shows that these complexes exhibit high thermal stability below 240 °C.

### UV-vis absorption spectra

**Fig. 2:**
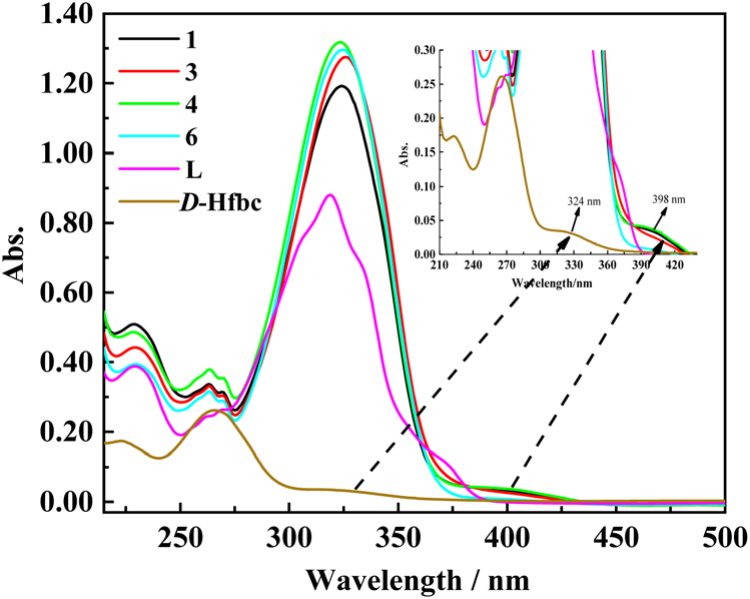
UV-vis absorption spectra of d-Hfbc, L and complexes 1, 3, 4, 6 in acetonitrile solutions.

As shown in Fig. 2, we obtained the UV–vis absorption spectra of ligand L, d-Hfbc, and complexes 1, 3, 4, and 6 in acetonitrile solutions at room temperature. The absorption peak of the ligand L at 319 nm can be attributed to the π–π* transition of the conjugated stilbene system of L. Because the ligand d-Hfbc has no conjugated system, the energy gap of the π–π* transition is relatively large and its maximum absorption peaks are at 265 and 223 nm. The maximum absorption peak for the n–π* transition of the C=O double bond in d-Hfbc is located at 324 nm. The UV–vis absorption spectra of complexes 1, 3, 4, 6, and L are similar, and the maximum absorption peaks for the π–π* transition of the complexes are all around 325 nm. Compared with that of the ligand L, the maximum absorption peaks of the four complexes are red-shift by 4–7 nm and the intensities are all stronger. This may be attributed to the charge transfer between lanthanide ions and ligands after coordination. The maximum absorption peak and molar extinction coefficients of the two ligands and four complexes are listed in Table 2. The four complexes all have strong absorption peaks around at 212–275 nm, showing superposition of the π–π* transition of ligands d-fbc^−^ and L. In addition, the four complexes present weak absorption peaks around 375–435 nm, which are due to the loss of one proton of Hfbc to coordinate with the lanthanide ion, resulting in a decrease in the n–π* transition energy of its carbonyl group.

**Table 2:** UV-vis absorption data of ligands and complexes 1, 3, 4, 6 in acetonitrile solutions

Compounds	*λ*_max_ [nm] (*ε*_max_ [10^4^ L mol^−1^ cm^−1^])			
Hfbc	319(4.40)	263(1.22)	229(1.93)	—
L	324(0.16)	265(1.30)	222(0.86)	—
1	324(5.96)	263(1.68)	229(2.54)	398(0.17)
3	326(6.38)	263(1.65)	229(2.21)	398(0.14)
4	323(6.59)	263(1.89)	229(2.43)	398(0.19)
6	325(6.48)	263(1.58)	229(1.97)	398(0.04)

### Electronic circular dichroism spectra

**Fig. 3:**
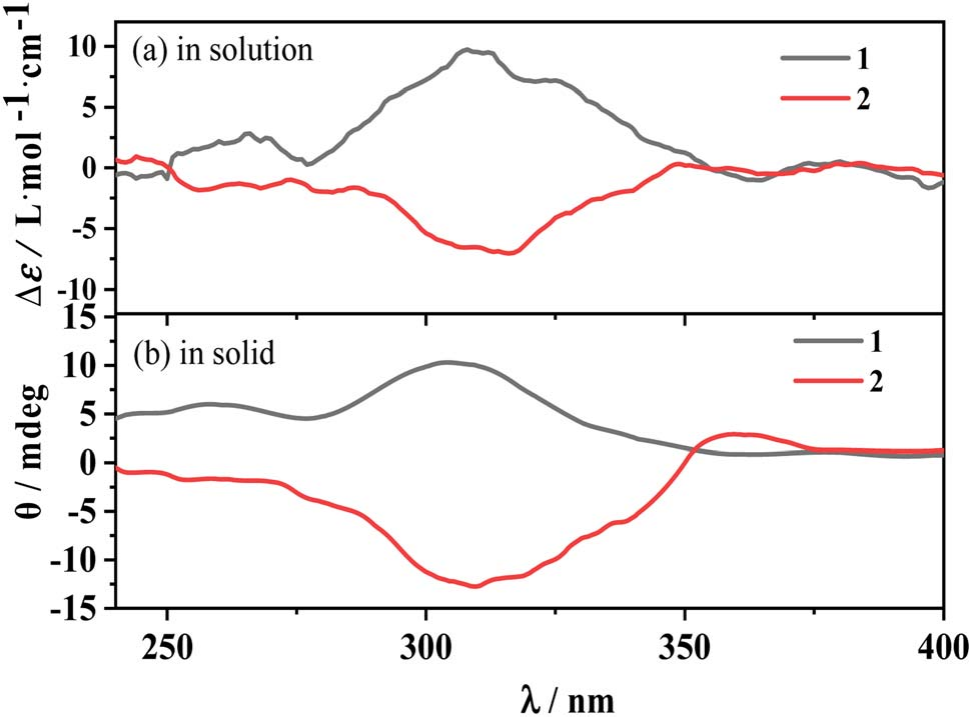
ECD spectra of complexes 1, 2 in acetonitrile solution (a) and in solid state (b), respectively.

The electronic circular dichroism (ECD) spectrum of d/l*-*Hfbc shows a peak at 265 nm originating from the π–π* transition of the ligand Hfbc. Since the four complexes have the same structure, only the enantiomers 1 and 2 were prepared and discussed. Fig. 3 shows the ECD spectra of complexes 1 and 2 in the solid state and in acetonitrile solutions at room temperature. Those of the others are shown in Fig. S4.† At 308 nm, complex 1 shows a positive ECD signal and complex 2 shows a negative ECD signal. Both ECD signals come from the chiral carbon of the ligand d/l*-*Hfbc, and there is no exciton splitting in the complexes in both solution and the solid state. The chiroptical signals originating from the Λ- and Δ-configurational chiralities of the two diastereomers in complex 1 or 2 should be diverse in magnitude and located at a slightly different wavelengths in the same region.^57,58^ However, the solid-state chiroptical spectra of complexes 1 and 2 are the same as the solution spectra without apparent bisignate couplets, indicating that the observed ECD spectra are a sum of the chiroptical signals from both diastereomers. Furthermore, the measured XRD pattern of complex 1 was found to be identical to the simulated data (Fig. S5†), which indicates that its bulk sample is a pure phase. This demonstrates the coexistence of the two absolute configurations of 1a and 1b or 2a and 2b in crystal 1 or 2 at a ratio of 1 : 1. According to the crystal data and the solution and solid-state spectra of complexes 3–6, they may have similar properties to those of complexes 1 and 2.

### Photoisomerization behavior of ligand L

**Fig. 4:**
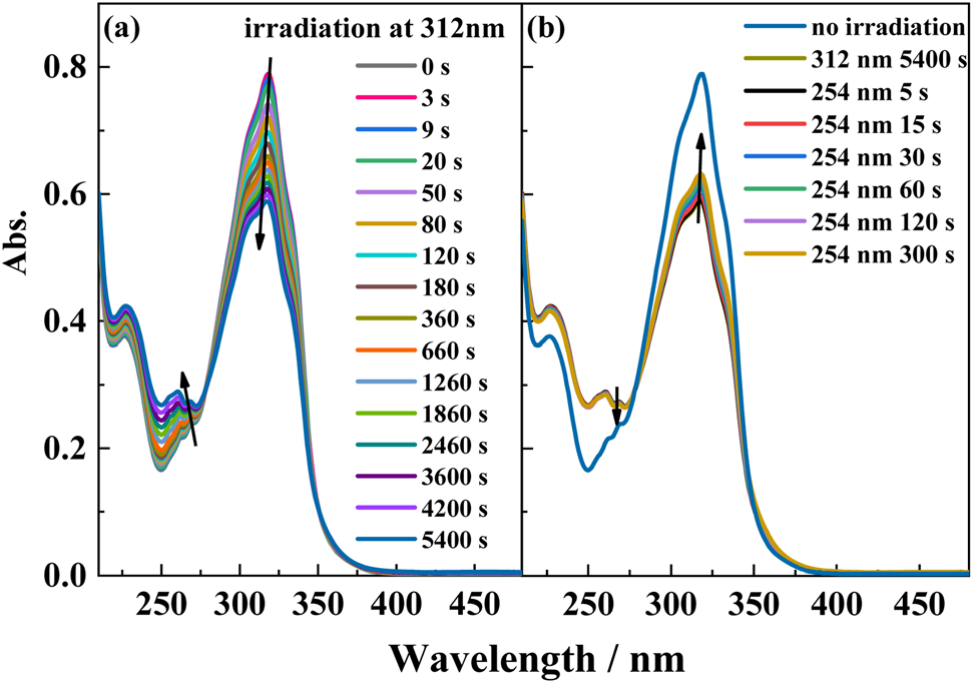
UV-Vis spectral change of L in acetonitrile solution (2.0 × 10^−5^ mol L^−1^) upon irradiation at UV-312 nm (a) and recoverable irradiation at UV-254 nm (b) as a function of time.

The reversible *trans*-to-*cis* photoisomerization of ligand L under UV irradiation at 312 and 254 nm was explored by UV–vis absorption spectroscopy. Under irradiation at 312 nm, the intensity of the maximum absorption peak for ligand L at 319 nm due to the π–π* transition of the conjugated stilbene system gradually decreases with increasing the illumination time. Because of steric hindrance, when ligand L isomerizes from the *trans* to the *cis* isomer, the two benzene rings cannot be coplanar, hence the conjugation effect is weakened, resulting in blue-shift of its absorption peak. Therefore, the intensity of the absorption peak at 319 nm is weakened, while the intensities of the absorption peaks at 229 and 262 nm are enhanced. Additionally, with the increase of illumination time, an isosbestic point appears at 277 nm, showing that the *trans*-to-*cis* photoisomerization reaction of ligand L occurs upon irradiation at 312 nm. Then, the solution was irradiated at 254 nm after it was irradiated at 312 nm for 1.5 h. The intensity of the absorption peak at 319 nm gradually increases with irradiation time at 254 nm, indicating that the *cis*-to-*trans* photoisomerization of L occurs under irradiation by 254 nm light (Fig. 4). From the variation of the UV–vis spectra with irradiation time, it can be calculated that 20% of the *cis* isomer is reconverted to the *trans* isomer after 5 min of irradiation, and no more *cis*-to-*trans* conversion occurs after 6 min irradiation. Then, the cyclability and reversibility of the isomerization of L were tested under alternate irradiation with 312 nm light for 3 min and 254 nm light for 5 min. The variation in the absorption intensity of L at 319 nm over 10 cycles is shown in Fig. S6.† With increasing cycle number, the absorption intensity of the ligand L gradually decreases and the cyclability gradually deteriorates. This is due to side reactions such as photocyclization and photodegradation occurring under UV light.

**Fig. 5:**
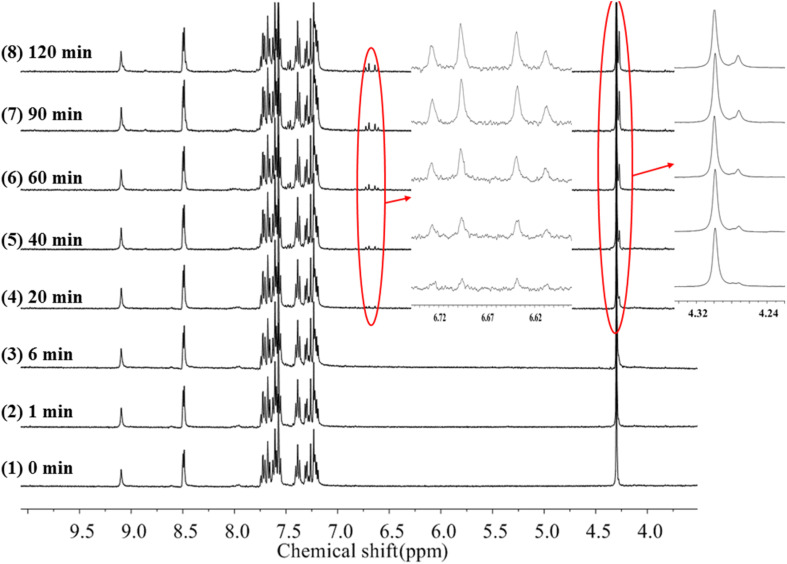
^1^H-NMR spectra changes of *trans*-L in acetonitrile-*d*_3_ irradiation at 312 nm light with increasing time.

**Fig. 6:**
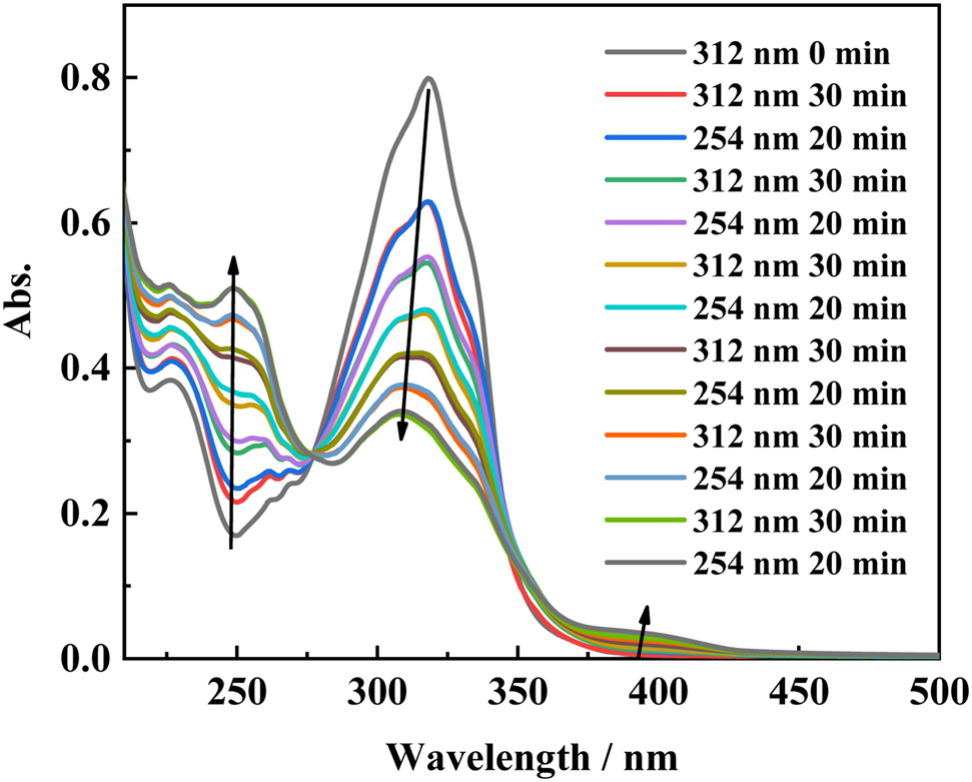
Absorption spectral changes of L in acetonitrile solution upon irradiation at UV-312 nm for 30 min, and UV-254 nm for 20 min alternatively.

To gain more insight into the side reactions of ligand L, we conducted ^1^H NMR spectra analysis at different irradiation times, as shown in Fig. 5. Under irradiation at 312 nm for the first 6 min, it is difficult to observe the changes of the proton signal in *trans* L. After the solution is irradiation for 20 min, new proton signals appear at 6.62–6.74 ppm, and the signal intensities gradually increase with irradiation time. Furthermore, a new peak at 4.27 ppm assigned to –CH_2_ also appears, indicating that a photochemical reaction occurs in the system when the solution is irradiated at 312 nm. Then, 254 nm light was used to continue irradiating the solution. However, under irradiation at 254 nm, the intensities of the peaks at 6.62–6.74 and 4.27 ppm remain almost unchanged. This indicates that after 2 h of illumination at 312 nm, the solution cannot recover its original state (Fig. S7†). Hence, after extended irradiation at 312 nm, the new proton signals at 6.62–6.74 ppm may be assigned to the proton signal for the C=C bond after *cis*-isomer cyclization to a dihydrophenanthrene group. Since the molecular formula of the photocyclization product is the same as that of L, the mass spectrum would not be changed under UV irradiation. This was confirmed by checking the mass-spectral variations for L in methanol solution with irradiation at 312 nm, as shown in Fig. S8.† Therefore, upon long-term UV irradiation, the side photoreaction is the photocyclization reaction. This can be confirmed by observing the changes in the photoisomerization first-order rate constants after extended irradiation under UV light. We calculated the photoisomerization first-order rate constants, and the apparent quantum yield of L was calculated by the same method reported previously (Table 3).^37^ The linear simulation of the first-order reaction kinetics for ligand L is good between 0 and 60 s and 300 and 2400 s, but no linear relationship is observed between 60 and 300 s (Fig. S9†). This indicates that the photocyclization reaction competes with the *trans*-to-*cis* photoisomerization between 60 and 300 s, while after 300 s, only photocyclization occurs. This can be further confirmed from the UV-spectral variations for L in acetonitrile solution upon alternating irradiation with UV light at 312 nm for 30 min and 254 nm for 20 min (Fig. 6). The intensity of the absorption peak at 319 nm gradually decreases with increasing irradiation time at 312 nm, while the intensities at 229 and 248 nm increase accompanied by the emergence of a new weak band centered at 395 nm.^59,60^ Appearance of the new absorption peak at 395 nm demonstrates the formation of the photocyclization product. Furthermore, the absorption intensities of these three peaks do not change upon irradiating the solution with 254 nm UV light, indicating that the *cis* isomer of L undergoes the photocyclization reaction and that the product of the photocyclization cannot be recovered to the *trans* isomer under 254 nm UV light.

**Table 3:** The photoisomerization rate constants *k*_iso_ (s^−1^) and quantum yields *Φ*_t–c_ of L and complexes 1, 3, 4, 6 in acetonitrile solutions

Compounds	10^3^*k*_iso_	10^2^*Φ*_t→c_
L	6.8	1.9
1	5.9	1.7
3	8.9	2.8
4	3.4	1.1
6	11.1	2.4

### Photoisomerization behaviors of complexes 1, 3, 4, and 6

**Fig. 7:**
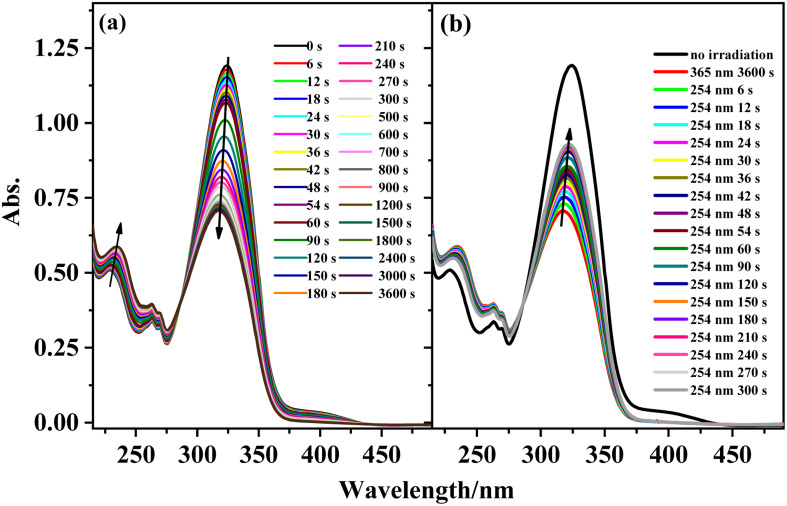
Absorption spectral changes of complex 1 in acetonitrile solution upon irradiation at UV-365 nm (a) and recoverable irradiation at UV-254 nm (b) as a function of time.

The photoisomerization properties of complexes 1, 3, 4, and 6 were examined (complexes 2 and 5 are enantiomers of complexes 1 and 4). The four complexes show similar photoisomerization behaviors under UV irradiation. Taking complex 1 as an example, Fig. 7 shows the variations in the UV–vis spectra of complex 1 under irradiation by 365 and 254 nm UV light as a function of time. With increasing irradiation time of the solution at 365 nm, the π–π* transition intensity of the stilbene moiety at 324 nm gradually decreases and shows a blue-shift of ∼7 nm, while the intensity of the peaks at 229 and 262 nm increase gradually with an isosbestic point appearing at 285 nm. This change is similar to that of the free ligand L. The reason for the blue-shift may be the charge–transfer transition between the metal center and the ligand after isomerization. Under irradiation at 254 nm, the UV–vis spectra of complex 1 shows the opposite change to that under irradiation at 365 nm. The absorption peak intensity of the π–π* transition for the stilbene moiety increases with irradiation time and exhibits a red-shift. Compared with L, complex 1 also shows similar photoisomerization behavior. There is a 53% conversion of the *cis* isomer to the *trans* isomer for complex 1 after 5 min irradiation at 254 nm. The cyclability and reversibility of complex 1 were investigated in the same way as those of ligand L. Although the intensity of the absorption peak at 324 nm gradually decreases during the cycling, the decrease in the intensity at 324 nm is much smaller than that of ligand L. After 10 cycles, complex 1 still shows excellent cyclability, proving that the complex has better photostability than that of ligand L. The cyclability of complex 1 and the photoisomerization properties of complexes 3, 4, and 6 are shown in Fig. S10 and S11.†

To further verify the photoisomerization properties of the complexes, the ^1^H NMR spectra of complex 1 at different irradiation times were obtained, as shown in Fig. S12 and S13.† Under UV irradiation at 365 nm, the signal of the proton peaks at 7.07–7.24 ppm attributed to the C=C bond of the *trans* isomer of complex 1 gradually weakens, while new proton signals at 6.52–6.74 ppm appear when the solution is irradiated at 365 nm for 5 min. Furthermore, the intensity of the proton signals at 6.52–6.74 ppm increases with irradiation at 365 nm, and no more changes occur after 2 h irradiation. Then, 254 nm light was used to irradiate the solution to study its recoverable photochemical reaction. Under UV irradiation at 254 nm, the intensity of the proton peaks at 6.52–6.74 ppm do not vary with irradiation time. Furthermore, the mass spectra do not change when the solution is irradiated under 365 nm UV light for more than 10 h. This indicates that upon long-term irradiation of the solution under UV light, the *cis* isomer of complex 1 also undergoes a photocyclization reaction. Owing to the paramagnetic nature of Sm^3+^, Eu^3+^, and Tb^3+^ ions, identifying the photoreactions by NMR spectral changes cannot be done. However, it can be assumed that complexes 3, 4, and 6 show similar results to those of complex 1 from the UV–vis spectral changes.

Furthermore, we calculated the photoisomerization first-order rate constants and the apparent quantum yields of the four complexes in acetonitrile solutions (Table 3). It was found that the first-order reaction kinetics of the complexes show similar properties and are a little different from that of pure L (Fig. S14†). Therefore, we only considered complex 1 as an example to discuss the reaction kinetics of the complexes in comparison with those of L. The rate constants for complex 1 are calculated for irradiation between 0 and 300 s. For irradiation between 0 and 300 s under 365 nm UV light, the linear fitting of the first-order reaction kinetics for complex 1 is good, with an *R* factor higher than 0.99 and a *trans*-to-*cis* photoisomerization rate constant calculated to be 5.9. When the irradiation time is extended beyond 300 s, the photoisomerization rate decreases gradually. This is due to photocyclization reactions competing with the *trans*-to-*cis* photoisomerization reaction. Like the situation for the free ligand L, the photocyclization reaction of complex 1 competes with the *trans*-to-*cis* photoisomerization between 300 and 900 s. After 900 s only the photocyclization reaction occurs. For ligand L, the linear fitting of the first-order reaction kinetics is only good between 0 and 80 s. This indicates that the *trans*-to-*cis* photoisomerization stability of the complex is higher than that of free L under 365 nm light irradiation. Compare with L, complexes 1, 3, 4, and 6 have appropriate photoisomerization rate constants (*k*_iso_) and photoisomerization quantum yields (*Φ*_t–c_) of 0.0059/0.017, 0.0089/0.028, 0.0034/0.011, and 0.0111/0.024, respectively, where those of complex 4 are the lowest. This is similar with our previous results for stilbene-attached europium complexes, where ligand-energy transfer to the Eu^3+^ ion suppressed stilbene photoisomerization.^47,48^

### Electronic absorption analysis

In order to further study the photophysical and photochemical properties of the complexes, we performed theoretical calculations on the ligands and complexes by DFT and TD-DFT. The structures of the ligands and complexes in acetonitrile were optimized. Table 4 shows the geometric parameters of the optimized complex 1 and its measured crystallographic data. The geometric parameters of complex 3 and its measured crystallographic data are shown in Table S6.† There is a small error between the structural geometric parameters optimized by R/UB3LYP and those measured by single crystal X-ray diffraction. Most of the theoretically calculated bond lengths are longer than the experimentally measured values, but the differences are within a reasonable and acceptable range. For complex 1, the experimentally measured average La–O bond length is 2.493 Å, La–N is 2.756 Å, and the stilbene C=C is 1.327 Å, while the calculated values (RB3LYP) are 2.510, 2.859, and 1.351 Å, respectively. For complex 3, the experimentally measured average Sm–O bond length is 2.420 Å, Sm–N is 2.679 Å, and the stilbene C=C is 1.310 Å, while the calculated values (UB3LYP) are 2.422, 2.774, and the stilbene C=C is 1.351 Å. The other complexes show similar results. The deviations may be ascribed to the fact that the theoretical calculation data for the complexes were obtained for acetonitrile solutions, and the experimental data were obtained for the solid. The solvation effect may cause the bonds between the lanthanide ions and the coordination atom to slightly elongate. The calculated bond lengths between the lanthanide ion and the coordination atom are close to the experimentally measured values, showing that the frozen core approximation assumed in the pseudopotential scheme is reasonable and that 4f electrons have negligible contributions to the bonding of lanthanide ions with coordinating atoms.

**Table 4:** The main structural geometric parameters obtained by the RB3LYP functional method for the optimization of complex 1 and the crystallographic data measured by single crystal X-ray diffractometer

Bond	Bond lengths (Å)		Bond	Bond angle (°)	
	measured	RB3LYP		measured	RB3LYP
La1–O1	2.535	2.591	O2–La1–N2	111.78	117.55
La1–O2	2.549	2.572	O6–La1–O2	74.62	72.97
La1–O3	2.436	2.440	O6–La1–O3	95.23	102.49
La1–O4	2.533	2.544	O6–La1–O4	76.14	76.27
La1–O5	2.479	2.494	O6–La1–O5	81.55	79.97
La1–O6	2.428	2.419	O6–La1–N2	135.02	132.36
La1–N1	2.718	2.812	O2–La1–N1	72.79	74.46
La1–N2	2.853	2.944	O3–La1–N2	129.38	124.97
La1–N3	2.697	2.821	O6–La1–N3	144.80	148.57
Trans C=C	1.327	1.351	N1–La1–N3	122.15	117.97

**Fig. 8:**
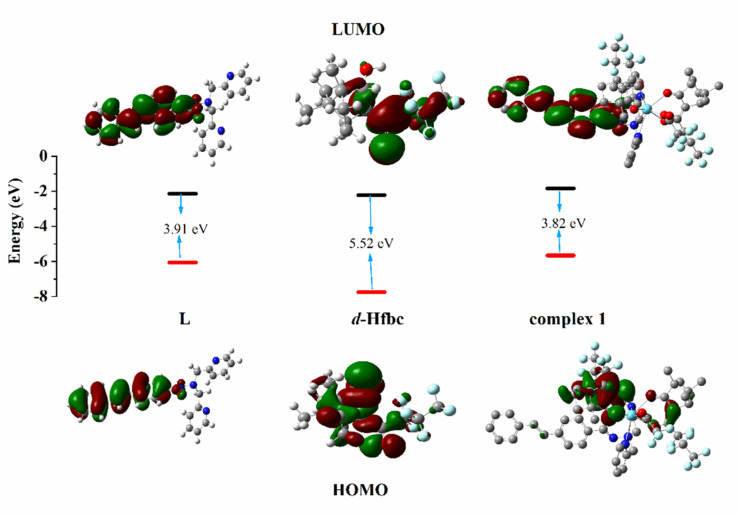
HOMO and LUMO energy levels and orbital distributions of L, d-Hfbc and complex 1. Most relevant MOs of complexes 1, 3, 4 and 6 associated with vertical excitation are shown in Table 5.

The calculated electron density distributions for the HOMOs and LUMOs of Hfbc, L, and complex 1 are shown in Fig. 8 (those of the other complexes are shown in Fig. S15†). The HOMO and LUMO electron density distributions of ligand L are mainly distributed on the stilbene groups and the amide bonds. The HOMO of Hfbc is mainly distributed on the camphor group and the carbonyl group, with a small fraction on the heptafluoropropyl group, while the LUMO is mainly distributed on the heptafluoropropyl group and the carbonyl group, with a small fraction on the camphor group. The four complexes have very different HOMO and LUMO electron density distributions. For complex 1, the HOMO is mainly distributed on one of fbc ligands, with a small fraction on the other fbc and the C=C double bond of L. The LUMO is mainly distributed on the stilbene group of L and the amide bond. The HOMO of complex 3 is mainly distributed on the stilbene group of L and one of the fbc ligands, with a small fraction on the amide bond of L, the other fbc, and the center Sm^3+^ ion. The LUMO orbital is mainly distributed on the stilbene group and the amide bond of ligand L and the center Sm^3+^ ion. The HOMO orbital of complex 4 is mainly distributed on one of the fbc ligands, with a small fraction on the other fbc. The LUMO orbital is mainly distributed on the stilbene group and the amide bond of the ligand L, with a small fraction on the center Eu^3+^. The HOMO orbital of complex 6 is mainly distributed on one of the fbc ligands, with a small fraction is on the other fbc, the center Tb^3+^ ion, and the amide group of L. The LUMO orbital is mainly distributed on the stilbene group and amide bond of L.

Based on the optimal geometries for the ground states of the four complexes, their electronic absorption spectra were calculated by TD-DFT. The implicit solvent model (SMD) was used to simulate the environment of complex 1 in acetonitrile. Fifty singlet states and 50 triplet states of the complex were selected, and six functional methods (B3LYP, CAM-B3LYP, WB97X, B3PW91, PBEPBE, and BPV86) were used for calculation.^47^ The obtained electron absorption spectra were compared with the UV–Vis spectra obtained experimentally. The electronic absorption spectra calculated by CAM-B3LYP are most similar to the UV–vis spectra measured experimentally (Fig. S16†). Therefore, the electronic absorption spectra of the other complexes were all calculated using CAM-B3LYP.

The main transitions with oscillator strengths *f* > 0.1 and dominant excitation characteristic of the four complexes are listed in Table 5. Compared with the UV–Vis spectra obtained by experiment, the maximum calculated peak has a slight blue-shift because the calculations cannot completely simulate the intermolecular forces of the complexes. The calculations show that the absorption bands in the range 230–400 nm are mainly derived from ligand-to-ligand charge transfer (LLCT) π_fbc_ → π_L*_ or π_L_ → π_fbc*_ transitions, intramolecular ligand (IL) π_L_ → π_L*_ and π_fbc_ → π_fbc*_ transitions, and ligand-to-metal ion charge transfer (LMCT) transitions. The maximal absorption (HOMO–LUMO transition) of the four simulated complexes are all located at 318 nm. Among them, the absorption peak of complex 1 is mainly attributed to charge transfer from the chiral ligand fbc to the ligand L (LLCT π_fbc_–π*_L_), with a small part attributed to the charge transition of the ligand L itself (IL π_L_ → π*_L_), which is shown by the molar extinction coefficient of the complex being higher than that of the ligand L. The charge transfer from ligand fbc to ligand L allows L to absorb more energy, so the stilbene group of ligand L in the complex is easier to isomerize, and the isomerization rate of complex 1 is higher than that of ligand L. For complex 3, the absorption peak at 318 nm is mainly attributed to LLCT π_fbc_–π*_L_, IL π_L_–π*_L_, and LMCT from L and fbc ligands to Sm^3+^ transitions. Complex 3 also shows a contribution from the IL π_L_–π*_L_ transition; thus the photoisomerization quantum yield and isomerization rate of complex 3 are higher than those of complex 1. For complex 4, except for LLCT π_fbc_–π*_L_, LMCT from fbc to Eu^3+^ has some contribution to the absorption peak at 318 nm. The LMCT transition reduces the energy obtained by ligand L, so the photoisomerization quantum yield and isomerization rate of complex 4 are lower than those of 1. The absorption peak at 318 nm for complex 6 is simply attributed to LLCT π_fbc_–π*_L_ transition, so the photoisomerization quantum yield and isomerization rate are similar to those of complex 3.

**Table 5:** Solvent-corrected (acetonitrile) major transition energies (eV) to excited states of complexes 1, 3, 4, 6 with contributing excitation (%), oscillator strengths (f), associated wavelengths (λ), and dominant excitation characters

Compounds	Transition	*f*	*E*/eV	*λ*/nm	Character
Complex 1	HOMO → LUMO (95%)	1.4993	3.90	318	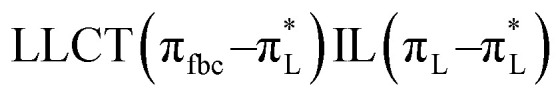
	H-2 → L+1 (19%) H-2 → L+2 (29%), H-1 → L+1 (29%)	0.1089	4.26	291	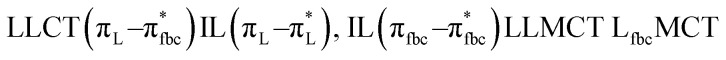
	H-2 → L+1 (43%) H-1 → L+2 (37%)	0.3562	4.34	286	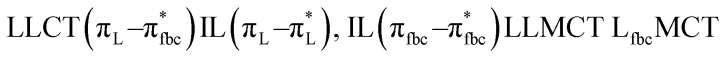
Complex 3	HOMO(A) → LUMO(A) (47%)	1.5080	3.90	318	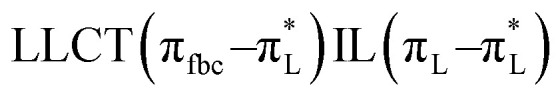 , L_L_MCT L_fbc_MCT
	H-2(A) → L+3(A) (18%),	0.1151	4.34	286	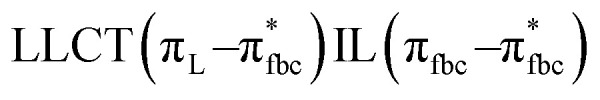 , L_L_MCT L_fbc_MCT
	H-1(A) → L+4(A) (30%)				
	H-2(A) → L+3(A) (52%),	0.1352	4.37	284	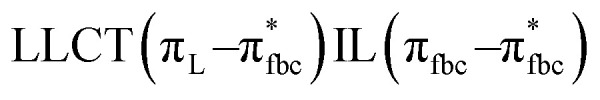 , L_L_MCT L_fbc_MCT
	H-1(A) → L+4(A) (11%)				
Complex 4	HOMO → LUMO (92%)	1.5064	3.90	318	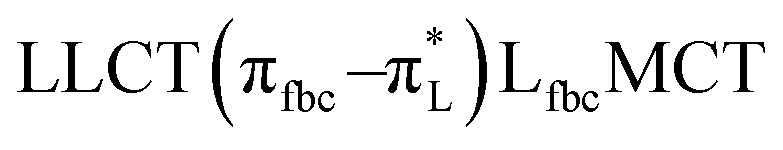
	H-2(A) → L+4(A) (45%)	0.2587	4.34	281	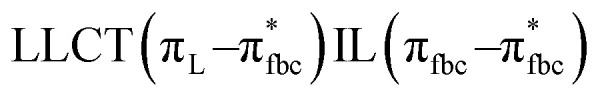 , L_L_MCT L_fbc_MCT L_HL_MCT
Complex 6	HOMO → LUMO (94%)	1.5142	3.90	318	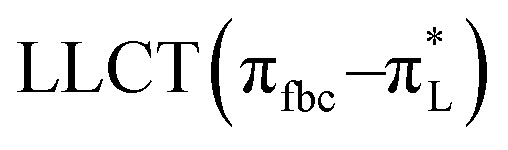
	H-4 → L+2 (19%) H-2 → L+1 (52%), H-2 → L+2 (13%)	0.1366	4.29	289	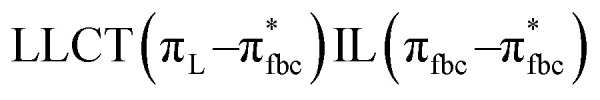 , L_L_MCT L_fbc_MCT
	H-4 → L+2 (21%) H-2 → L+1 (11%), H-1 → L+1 (10%) H-1 → L+2 (40%)	0.2208	4.35	285	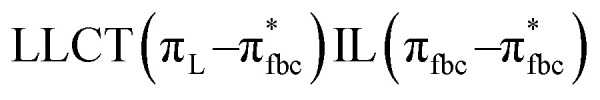 , L_L_MCT L_fbc_MCT

### Luminescence emission analysis

**Fig. 9:**
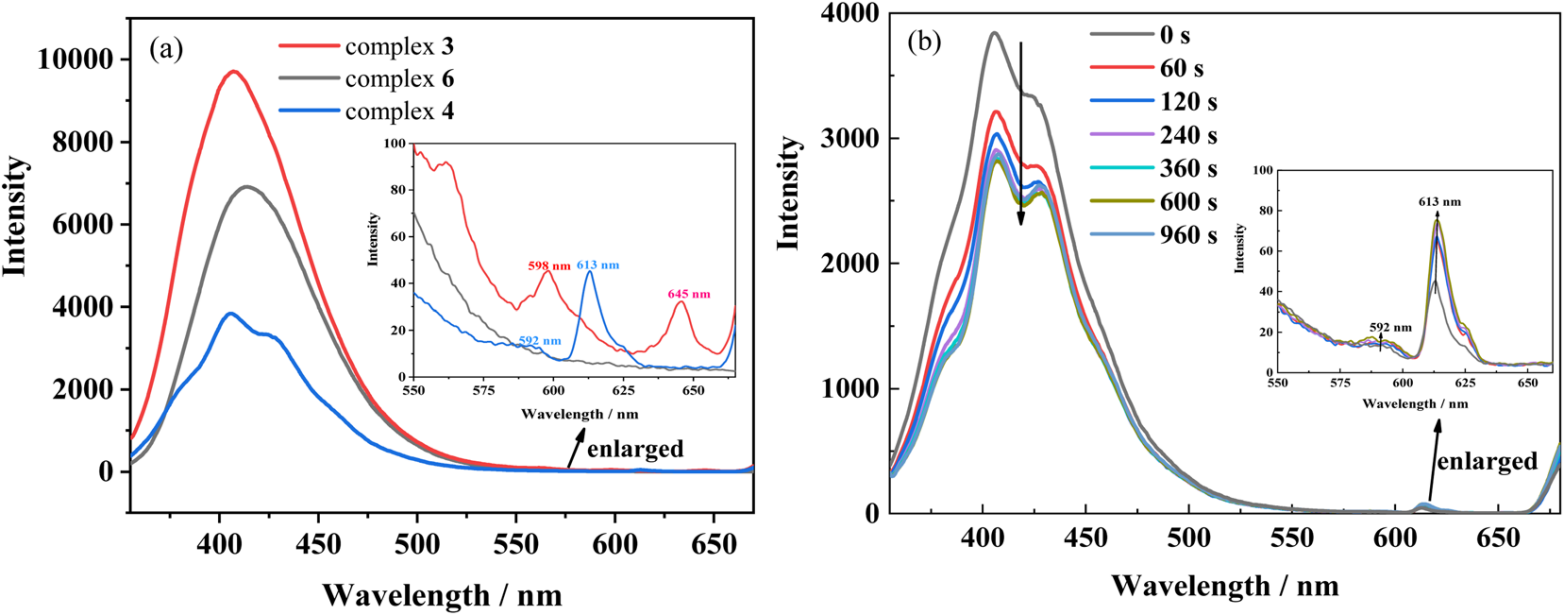
(a) Luminescence spectra of complexes 3, 4 and 6 in acetonitrile solution (2.0 × 10^−5^ mol L^−1^) (b) luminescence spectral changes of complex 4 upon irradiation at UV-365 nm as a function of time. Inset shows the enlarged luminescence images in the range of 550 ∼680 nm wavelength. (*λ*_ex_ = 340 nm).

In addition, the first excited singlet and triplet levels of ligand Hfbc and the *cis* and *trans* isomers of L were calculated by TD-DFT. A comparison between the first excited triplet levels of the ligands and the minimum excitation energy levels of the lanthanide ions Sm^3+^, Eu^3+^, and Tb^3+^ are shown in Fig. S17.† The lowest excited triplet energy levels of Hfbc and the *trans* and *cis* isomers of L are 28 127, 18 131, and 21 016 cm^−1^, respectively. The lowest excited triplet energy level of Hfbc is higher than those of Sm^3+^ (^4^G_5/2_, 17 762 cm^−1^), Eu^3+^ (^5^D_0_, 17 241 cm^−1^), and Tb^3+^ (^5^D_4_, 20 496 cm^−1^), for which the differences are 10 365, 10 886, and 7631 cm^−1^, respectively. Thus, the energy level differences are beyond the range 2500–3500 cm^−1^.^37^ Therefore, although the ligand Hfbc can transfer energy to Sm^3+^, Eu^3+^, and Tb^3+^ ions, the sensitization efficiency is low. The lowest excited triplet energy level of the *trans* isomer of L is higher than those of Sm^3+^ (^4^G_5/2_, 17 762 cm^−1^) and Eu^3+^ (^5^D_0_, 17 241 cm^−1^), and the differences are 369 and 890 cm^−1^, respectively. These energy level differences are also not in the range 2500–3500 cm^−1^, so the energy of the *trans* isomer of L does not readily transfer to Sm^3+^ and Eu^3+^ ions. Moreover, owing to the chirality and weak conjugation of the ligand Hfbc, the sensitization efficiency is also reduced. The sensitization efficiencies for the ligands L and Hfbc are both low, so only the weak characteristic 4f–4f luminescence at 598 nm (4G_5/2_ → 6H_7/2_) and 645 nm (4G_5/2_ → 6H_9/2_) transitions for complex 3 and the 592 nm (5D_0_ → 7F_1_) and 613 nm (5D_0_ → 7F_2_) transitions for complex 4 are observed (Fig. 9). Complex 6 mainly emits the luminescence of ligand L and no characteristic 4f–4f luminescence is observed. However, the energy level differences between ligand L as the *cis* isomer and the lanthanide ions Sm^3+^ (^4^G_5/2_, 17 762 cm^−1^), Eu^3+^ (^5^D_0_, 17 241 cm^−1^), Tb^3+^ (^5^D_4_, 20 496 cm^−1^), are 3254, 3775, and 520 cm^−1^, of which the energy level difference for Sm^3+^ (3254 cm^−1^) is in the range 2500–3500 cm^−1^ and the energy level difference for Eu^3+^ (3775 cm^−1^) is close to the range of 2500–3500 cm^−1^. Thus, the *cis* isomer ligand L is assumed to sensitize Sm^3+^and Eu^3+^. Accordingly, the luminescence spectra of complexes 3 and 4 were obtained for different durations of UV irradiation at 365 nm. The results show that luminescence intensity around 350–500 nm originated from the ligand transition of complex 3 decreases with increasing irradiating time, but the increases in luminescence intensity at 598 nm (4G_5/2_ → 6H_7/2_) and 645 nm (4G_5/2_ → 6H_9/2_) are difficult to observe owing to the very weak signal. For complex 4, the characteristic 4f–4f luminescence intensities at 592 nm (5D_0_ → 7F_1_) and 613 nm (5D_0_ → 7F_2_) increase with irradiation time during the *trans*-to-*cis* photoisomerization accompanied by a decrease in intensity of the ligand luminescence around 350–500 nm. Although the energy level difference between ligand L as the *cis* isomer and the lanthanide ions Sm^3+^ and Eu^3+^ are relatively close to the 2500–3500 cm^−1^ range, sensitization by *cis* isomer of ligand L of the Sm^3+^and Eu^3+^ luminescence efficiency is still low. This may be attributed to the low site symmetry of the lanthanide ions in the complexes. In addition, the luminescences of complexes 3 and 4 are cyclable and reversible under alternate irradiation with 365 nm light for 2 min and 254 nm light for 2 min. The variation of luminescence intensity for more than 10 cycles is shown in Fig. S18.† This indicates that rationally designed stilbene-functionalized lanthanide complexes are potential lanthanide-based optical-switching materials.

## Conclusions

Six new chiral di-β-diketonate (d/l*-*fbc = 3-heptafluorobutyryl-(+)/(−)-camphorate) lanthanide (La^3+^, Sm^3+^, Eu^3+^, and Tb^3+^) complexes containing a stilbene derivative (*E*)-*N*′,*N*′-bis(pyridin-2-ylmethyl)-4-styrylbenzohydrazide were synthesized and characterized. Four crystals of the complexes were obtained and analyzed. The asymmetric unit within the unit cell consists of two independent molecules and two water molecules. The two independent molecules in diastereomeric Δ and Λ forms coexist in the crystals. Complex 4 shows the smallest photoisomerization rate constant (*k*_iso_) and quantum yield (*Φ*_t–c_) among the complexes. Furthermore, complexes 3 and 4 show reversible luminescence change upon *trans-*to-*cis* photoisomerization during their photochemical reactions. However, the photocyclization reaction is followed by a *trans*-to-*cis* photoisomerization reaction that competes with the *trans*-to-*cis* photoisomerization, then the photocyclization reaction continues to occur. The photocyclization reaction is unreversible in this stilbene derivative but delayed in the lanthanide complexes. Such complexes show superior properties to those of pure stilbene for molecular-switching applications. Thorough study of the luminescence, *trans*-to-*cis* photoisomerization, and photocyclization of the complexes indicated that the dual functionalities of the lanthanide complexes may open a route to rationally designed functional smart stilbene-based materials for optical-switching applications.

## Conflicts of interest

There are no conflicts to declare.

## Author contributions

L.-R. Lin supervised the project, rewrote, edited and revised the paper. Z. Hou, Y. Ruan and Y. Tan carried out the experiments. H. Xu helped to characterize the crystal structures. Y. Huang wrote the original draft and carried out some experiments. Z. Wu helped funds. All authors discussed the results and assisted manuscript preparation.

## Supplementary Material

RA-013-D2RA07133A-s001

RA-013-D2RA07133A-s002

## References

[cit1] Xu K., Xie X. J., Zheng L. M. (2022). Iridium-lanthanide complexes: Structures, properties and applications. Coord. Chem. Rev..

[cit2] Luo Z. C., Liang X. Q., He T., Qin X., Li X. C., Li Y. S., Li L., Loh X. J., Gong C. Y., Liu X. G. (2022). Lanthanide-nucleotide coordination nanoparticles for STING activation. J. Am. Chem. Soc..

[cit3] Bao G. C., Wen S. H., Lin G. G., Yuan J. L., Lin J., Wong K. L., Bunzli J. C. G., Jin D. Y. (2021). Learning from lanthanide complexes: The development of dye-lanthanide nanoparticles and their biomedical applications. Coord. Chem. Rev..

[cit4] Zou Q., Huang X. D., Liu J. C., Bao S. S., Zheng L. M. (2019). Lanthanide anthracene complexes: slow magnetic relaxation and luminescence in Dy-III, Er-III and Yb-III based materials. Dalton Trans..

[cit5] Wei C., Ma L., Wei H. B., Liu Z. W., Bian Z. Q., Huang C. H. (2018). Advances in luminescent lanthanide complexes and applications. Sci. China: Technol. Sci..

[cit6] Mikami K., Terada M., Matsuzawa H. (2002). Asymmetric catalysis by lanthanide complexes. Angew. Chem., Int. Ed..

[cit7] Inanaga J., Furuno H., Hayano T. (2002). Asymmetric catalysis and amplification with chiral lanthanide complexes. Chem. Rev..

[cit8] Shibasaki M., Yoshikawa N. (2002). Lanthanide complexes in multifunctional asymmetric catalysis. Chem. Rev..

[cit9] Eliseeva S. V., Bunzli J. C. G. (2010). Lanthanide luminescence for functional materials and bio-sciences. Chem. Soc. Rev..

[cit10] Kovalenko A., Rublev P. O., Tcelykh L. O., Goloveshkin A. S., Lepnev L. S., Burlov A. S., Vashchenko A. A., Marciniak L., Magerramov A. M., Shikhaliyev N. G., Vatsadze S. Z., Utochnikova V. V. (2019). Lanthanide complexes with 2-(Tosylamino)-benzylidene-N-(aryloyl)hydrazones: Universal luminescent materials. Chem. Mater..

[cit11] Wilharm R. K., Raju M. V. R., Hoefler J. C., Platas-Iglesias C., Pierre V. C. (2022). Exploiting the fluxionality of lanthanide complexes in the design of paramagnetic fluorine probes. Inorg. Chem..

[cit12] Ambiliraj D. B., Francis B., Reddy M. L. P. (2022). Lysosome-targeting luminescent lanthanide complexes: from molecular design to bioimaging. Dalton Trans..

[cit13] Li P., Li H. R. (2021). Recent progress in the lanthanide-complexes based luminescent hybrid materials. Coord. Chem. Rev..

[cit14] Zhang W. Y., Liang H. D., Qin X. Y., Yuan J. M., Wang X., Wang Z. G., Wang Y., Zhang J. C., Yang D. Q. (2022). Double-network luminescent films constructed using sulfur quantum dots and lanthanide complexes. ACS Appl. Mater. Interfaces.

[cit15] Abu-Yamin A. A., Abduh M. S., Saghir S. A. M., Al-Gabri N. (2022). Synthesis, characterization and biological activities of new Schiff base compound and its lanthanide complexes. Pharmanual.

[cit16] da Rosa P. P. F., Kitagawa Y., Shoji S., Oyama H., Imaeda K., Nakayama N., Fushimi K., Uekusa H., Ueno K., Goto H., Hasegawa Y. (2022). Preparation of photonic molecular trains via soft-crystal polymerization of lanthanide complexes. Nat. Commun..

[cit17] Song B., Wen X. Y., Zhang X. Y., Liu Q., Ma H., Tan M. Q., Yuan J. L. (2021). Bioconjugates of versatile beta-diketonate-lanthanide complexes as probes for time-gated luminescence and magnetic resonance imaging of cancer cells in vitro and in vivo. J. Mater. Chem. B.

[cit18] Rodrigues C. V., Johnson K. R., Lombardi V. C., Rodrigues M. O., Sobrinho J. A., de Bettencourt-Dias A. (2021). Photocytotoxicity of thiophene- and bithiophene-dipicolinato luminescent lanthanide complexes. J. Med. Chem..

[cit19] Martin K. E., Cosby A. G., Boros E. (2021). Multiplex and in vivo optical imaging of discrete luminescent lanthanide complexes enabled by in situ cherenkov radiation mediated energy transfer. J. Am. Chem. Soc..

[cit20] Leguerrier D. M. D., Barre R., Molloy J. K., Thomas F. (2021). Lanthanide complexes as redox and ROS/RNS probes: A new paradigm that makes use of redox-reactive and redox non-innocent ligands. Coord. Chem. Rev..

[cit21] Bodman S. E., Butler S. J. (2021). Advances in anion binding and sensing using luminescent lanthanide complexes. Chem. Sci..

[cit22] Lan J. F., Li J., Zhu J. L., Yan G. P., Ke H., Liao J. Z. (2021). Radical-doped crystalline lanthanide-based photochromic complexes: Self-assembly driven by multiple interactions and photoswitchable luminescence. Inorg. Chem..

[cit23] Lin J. F., Wang P., Wang H. J., Shi Y. J., Zhu K., Yan F., Li G. H., Ye H. H., Zhai J. W., Wu X. (2021). Significantly photo-thermochromic KNN-based Smart Window for sustainable optical data storage and anti-counterfeiting. Adv. Opt. Mater..

[cit24] Verma P., Singh A., Maji T. K. (2021). Photo-modulated wide-spectrum chromism in Eu^3+^ and Eu^3+^/Tb^3+^ photochromic coordination polymer gels: application in decoding secret information. Chem. Sci..

[cit25] Kocsi D., Orthaber A., Borbas K. E. (2022). Tuning the photophysical properties of luminescent lanthanide complexes through regioselective antenna fluorination. Chem. Commun..

[cit26] Hasegawa M., Ohmagari H., Tanaka H., Machida K. (2022). Luminescence of lanthanide complexes: From fundamental to prospective approaches related to water- and molecular-stimuli. J. Photochem. Photobiol., C.

[cit27] Lin J. F., Zhou Y., Lu Q. L., Wu X., Lin C., Lin T. F., Xue K. H., Miao X. S., Sa B. S., Sun Z. M. (2019). Reversible modulation of photoenergy in Sm-doped (K0.5Na0.5)NbO3 transparent ceramics via photochromic behavior. J. Mater. Chem. A.

[cit28] Zhou W. L., Chen Y., Yu Q. L., Li P. Y., Chen X. M., Liu Y. (2019). Photo-responsive cyclodextrin/anthracene/Eu^3+^ supramolecular assembly for a tunable photochromic multicolor cell label and fluorescent ink. Chem. Sci..

[cit29] Zhou Y., Zhang H. Y., Zhang Z. Y., Liu Y. (2017). Tunable luminescent lanthanide supramolecular assembly based on photoreaction of anthracene. J. Am. Chem. Soc..

[cit30] Jeong W., Khazi M. I., Park D. H., Jung Y. S., Kim J. M. (2016). Full color light responsive diarylethene inks for reusable paper. Adv. Funct. Mater..

[cit31] Nishi H., Namari T., Kobatake S. (2011). Photochromic polymers bearing various diarylethene chromophores as the pendant: synthesis, optical properties, and multicolor photochromism. J. Mater. Chem..

[cit32] Yang Y. H., Li Y. Q., Chen Y. L., Wang Z. H., He Z., He J. Z., Zhao H. M. (2022). Dynamic anticounterfeiting through
novel photochromic spiropyran-based switch@Ln-MOF composites. ACS Appl. Mater. Interfaces.

[cit33] Zhang H. B., Chen Z. H., He Y. R., Yang S. Y., Wei J. (2021). Spiropyran-modified upconversion nanoparticles for tunable fluorescent printing. ACS Appl. Nano Mater..

[cit34] Zhao Y. Q., Li Z., Ma J. T., Jia Q. (2021). Design of a spiropyran-based smart adsorbent with dual response: Focusing on highly efficient enrichment of phosphopeptides. ACS Appl. Mater. Interfaces.

[cit35] Li Q. F., Xia H. J., Li E., Wang J. T., Wang Z. L. (2022). Photocontrolled reversible modulation of lanthanide luminescence in mesoporous silica nanospheres by photochromic diarylethenes. J. Mater. Chem. C.

[cit36] Al Sabea H., Norel L., Galangau O., Roisnel T., Maury O., Riobe F., Rigaut S. (2020). Efficient photomodulation of visible Eu(III) and invisible Yb(III) luminescences using DTE photochromic ligands for optical encryption. Adv. Funct. Mater..

[cit37] Lin L. R., Tang H. H., Wang Y. G., Wang X., Fang X. M., Ma L. H. (2017). Functionalized lanthanide(III) complexes constructed from azobenzene derivative and beta-diketone ligands: Luminescent, magnetic, and reversible trans-to-cis photoisomerization properties. Inorg. Chem..

[cit38] Wang Y. G., Li Y. Q., Tang H. H., Lin L. R., Ma L. H. (2018). Near-infrared photoluminescence and reversible trans-to-cis photoisomerization of mononuclear and binuclear ytterbium(III) complexes functionalized by azobenzene groups. ACS Omega.

[cit39] Fu C. Y., Chen L., Wang X., Lin L. R. (2019). Synthesis of bis-beta-diketonate lanthanide complexes with an azobenzene bridge and studies of their reversible photo/thermal isomerization properties. ACS Omega.

[cit40] Waldeck D. H. (1991). Photoisomerization Dynamics of Stilbenes. Chem. Rev..

[cit41] Mukherjee S., Sahoo A., Deb S., Baitalik S. (2021). Light and cation-driven optical switch based on a stilbene-appended terpyridine system for the design of molecular-scale logic devices. J. Phys. Chem. A.

[cit42] Al-Busaidi I. J., Haque A., Husband J., Al Rasbi N. K., Abou-Zied O. K., Al Balushi R., Khan M. S., Raithby P. R. (2021). Electronic and steric effects of platinum(II) di-yne and poly-yne substituents on the photo-switching behaviour of stilbene: experimental and theoretical insights. Dalton Trans..

[cit43] Villaron D., Wezenberg S. J. (2020). Stiff-stilbene photoswitches: From fundamental studies to emergent applications. Angew. Chem., Int. Ed..

[cit44] Liu F. Y., Morokuma K. (2012). Computational study on the working mechanism of a stilbene light-driven molecular rotary motor: Sloped minimal energy path and unidirectional nonadiabatic photoisomerization. J. Am. Chem. Soc..

[cit45] Wei C. Y., Zhang W. F., Huang J. Y., Li H., Zhou Y. K., Yu G. (2019). Realizing n-Type Field-Effect Performance via Introducing Trifluoromethyl groups into the donor acceptor copolymer backbone. Macromolecules.

[cit46] Frullano L., Zhu J., Miller R. H., Wang Y. (2013). Synthesis and characterization of a novel gadolinium-based contrast agent for magnetic resonance imaging of myelination. J. Med. Chem..

[cit47] Chen L., Tan Y., Xu H., Wang K., Chen Z. H., Zheng N., Li Y. Q., Lin L. R. (2020). Enhanced E/Z-photoisomerization and luminescence of stilbene derivative co-coordinated in di-beta-diketonate lanthanide complexes. Dalton Trans..

[cit48] Xu H., Tan Y., Hou Z., Fu C., Lin L. R. (2022). Insights into the effect of trans-to-cis photoisomerization of a co-coordinated stilbene derivative on the luminescence of di-beta-diketonate lanthanide
complexes. ACS Omega.

[cit49] Sheldrick G. M. (2015). SHELXT - integrated space-group and crystal-structure determination. Acta Crystallogr., Sect. A: Found. Adv..

[cit50] Sheldrick G. M. (2015). Crystal structure refinement with SHELXL. Acta Crystallogr., Sect. C: Struct. Chem..

[cit51] Dolomanov O. V., Bourhis L. J., Gildea R. J., Howard J. A. K., Puschmann H. (2009). OLEX2: a complete structure solution, refinement and analysis program. J. Appl. Crystallogr..

[cit52] Becke A. D. (1993). Density-functional thermochemistry .3. The role of exact exchange. J. Chem. Phys..

[cit53] Ramanantoanina H. (2018). A DFT-based theoretical model for the calculation of spectral profiles of lanthanide M_4,5_-edge x-ray absorption. J. Chem. Phys..

[cit54] Dolg M., Stoll H., Preuss H. (1989). Energy-adjustedabinitiopseudopotentials for the rare earth elements. J. Chem. Phys..

[cit55] Cao X. Y., Dolg M. (2002). Segmented contraction scheme for small-core lanthanide pseudopotential basis sets. J. Mol. Struct.: THEOCHEM.

[cit56] O'Boyle N. M., Tenderholt A. L., Langner K. M. (2008). cclib: a library for package-independent computational chemistry algorithms. J. Comput. Chem..

[cit57] Lin Y. J., Zou F., Wan S. G., Ouyang J., Lin L. R., Zhang H. (2012). Dynamic chiral-at-metal stability of tetrakis(d/l*-*hfc)Ln(III) complexes capped with an alkali metal cation in solution. Dalton Trans..

[cit58] Lin Y. J., Wan S. G., Zou F., Wang Y. K., Zhang H. (2011). Structure-chiroptical property relationship of kinetically labile camphor-derivative beta-diketone Yb(III) complexes: do the adducts coexist as diastereomers or not?. New J. Chem..

[cit59] Mustkat K. A., Fischer E. (1967). Structure, spectra, photochemistry, and thermal reactions of the 4a, 4b-Dihydrophenanthrenes. J. Chem. Soc. B.

[cit60] Doyle T. D., Benson W. R., Filipescu N. (1976). Photocyclization of diethylstilbestrol. isolation of a stable, self-Trapping dihydrophenanthrene intermediate. J. Am. Chem. Soc..

