# Supplementary material for: Reversible trans-to-cis photoisomerization and irreversible photocyclization reactions of a Co-coordinated stilbene derivative on chiral di-β-diketonate lanthanide complexes
Source: RSC Adv. 2023 Jan 13;13(4):2269–82. doi: 10.1039/d2ra07133a (PMC9837704; doi:10.1039/d2ra07133a)
Supplement: RA-013-D2RA07133A-s001 [file RA-013-D2RA07133A-s001.pdf]

Electronic Supplementary Material (ESI) for RSC Advances.
This journal is © The Royal Society of Chemistry 2023

Reversible trans-to-*cis* Photoisomerization and Unreversible Photocyclization Reactions of a Co-coordinated Stilbene Derivative on the Chiral di-β-Diketonate Lanthanide Complexes

Ziting Hou[1], Yanji Huang[1#], Yushan Ruan[1#], Han Xu[2], Yu Tan[1], Li-Rong Lin[1]*, Zhen-yi Wu[1]

[1] Department of Chemistry, College of Chemistry and Chemical Engineering, Xiamen University, Xiamen, 361005, P. R. China

[2] School of Pharmaceutical Science & Yunnan Key Laboratory of Pharmacology for Natural Products, Kunming Medical University, Kunming 650500, P. R. China

Contents

Table S1. Selected bond lengths (Å) for complexes **1** and **2**. 3
Table S2. Selected bond lengths (Å) for complexes **3** and **4**. 4
Table S3. Selected angles (deg) of complexes **1** and **2**. 5
Table S4. Selected angles (deg) of complexes **3** and **4** 6
Table S5. Hydrogen-bonding Geometry (Å and °) 7
Figure S1. The polyhedron geometry of center La(III) of complexes **1** (1a, 1b, left) and **2** (2a, 2b, right). 7
Figure S2. The Crystal structure and coordination polyhedron geometry of the two independent molecules of complexes **3** (3-1, 3-1a in left) and **4** (4-1, 4-1a in right) in the unit cell. 8
Figure S3. Thermogravimetric analysis (TGA) curves of complexes **1** (black), **3** (red), **4** (blue) and **5** (magenta). 9
Figure S4. ECD spectra of *d*/l-Hfbc, complexes **4**, **5** (a) and complexes **3**, **6** (b) in acetonitrile solution, and complexes **4**, **5** (c) and **3**, **6** (d) in solid state, respectively. 9
Figure S5. Observed (red) and calculated (black) X-ray powder diffraction patterns of complexes **1** (a) and **3** (b). 10
Figure S6. Cycles of maximum absorption intensity at 319 nm of ligand L in acetonitrile solutions are modulated by the irradiation of UV light at 365 nm for 10 min and 254 nm for 5 min, alternatively. 10
Figure S7. Evolution of ^{1}H-NMR spectra of L in acetonitrile-d_3 solution (after 365 nm light irradiation for 2 h) at 254 nm light with increasing irradiation time. 11
Figure S8. Mass spectra of L in methanol solution exposed to 312 nm UV light in different irradiation time. 12
Figure S9.The *trans-cis* photoisomerization and photocyclization reactional kinetics of $\ln(A_\infty - A_0)/(A_\infty - A_t)$ as a function of time for L in acetonitrile solution. 13

Figure S10. Cycles of maximum absorption intensity at 324 nm of complex 1 in acetonitrile solution are modulated by the irradiation of UV light at 365 nm for 3 min and 254 nm for 5 min, alternatively..14
Figure S11. UV-Vis spectral change of complexes **3** (a, b), **4** (e, f), 6 (h, j) in acetonitrile solutions (2.0×10^{-5} mol·L^{-1}) upon irradiation at UV-365 nm and recoverable irradiation at UV-254 nm as a function of time and cycles of maximum absorption intensity at 324 nm are modulated by the irradiation of UV light at 365 nm for 3 min and 254 nm for 5 min, alternatively (c for complex **3**, g for complex **4**, k for complex **6**). 15
Figure S12. Evolution of ^{1}H-NMR spectra of complex **1** in acetonitrile-d_3 solution at 365 nm light with increasing irradiation time. 16
Figure S13. Evolution of ^{1}H-NMR spectra of complex 1 in acetonitrile-d_3 solution (after 365 nm light irradiation for 2 h) at 254 nm light with increasing irradiation time....................................17
Figure S14.The *trans-cis* photoisomerization kinetics of $\ln(A\infty-A_0)/((A\infty-A_t))$ as a function of time for complexes **1** (a, b, c), **3** (d, e, f), **4** (g, h, i), and **6** (j, k, l) in acetonitrile solutions..........18
Table S6. The main structural geometric parameters obtained by the RB3LYP functional method for the optimization of complex **3** and the crystallographic data measured by single crystal X-ray diffractometer...19
Figure S15. HOMO and LUMO energy levels and orbital distributions of complexes **3**, **4** and **6**. 20
Figure S16. Overlay of calculated and experimental absorption spectra of the complex **1** in acetonitrile solutions. 21
Figure S17. The energy levels of the first excited states of ligands *trans*-L, *cis*-L, Hfbc and the related energy levels of the Sm^{3+}, Eu^{3+} and Tb^{3+}. 22
Figure S18. Cycles of maximum luminescence intensity at 420 nm are modulated by the irradiation of UV light at 365 nm for 2 min and 254 nm for 2 min, alternatively (a for complex 3, b for complex 4)...22

Table S1. Selected bond lengths (Å) for complexes 1 and 2.

Complex 1 $[La(\textit{d}-fbc)_2(L)(CF_3COO)]\cdot H_2O$		Complex 2 $[La(\textit{l}-fbc)_2(L)(CF_3COO)]\cdot H_2O$	
**Bond**	**lengths (Å)**	**Bond**	**lengths (Å)**
La1−O1	2.535(3)	La1−O1	2.547(7)
La1−O2	2.549(3)	La1−O2	2.525(7)
La1−O3	2.436(3)	La1−O3	2.449(7)
La1−O4	2.533(3)	La1−O4	2.503(7)
La1−O5	2.479(3)	La1−O5	2.498(7)
La1−O6	2.428(3)	La1−O6	2.445(8)
La1−N1	2.718(4)	La1−N1	2.684(9)
La1−N2	2.853(4)	La1−N2	2.841(10)
La1−N3	2.697(3)	La1−N3	2.730(8)
La2−O9	2.538(3)	La2−O9	2.533(7)
La2−O10	2.459(3)	La2−O10	2.445(6)
La2−O11	2.529(3)	La2−O11	2.545(7)
La2−O12	2.485(3)	La2−O12	2.480(7)
La2−O13	2.499(3)	La2−O13	2.531(7)
La2−O14	2.442(3)	La2−O14	2.421(7)
La2−N5	2.681(4)	La2−N5	2.733(8)
La2−N6	2.839(4)	La2−N6	2.845(9)
La2−N7	2.719(4)	La2−N7	2.711(8)

* O2, O3 and O4, O5 are from the two *d*-fbc, O6 is from trifluoroacetate, O1 and N2 are from benzohydazide and N1, N3 are from pyridines of ligand L.

Table S2. Selected bond lengths (Å) for complexes 3 and 4.

Complex 3 $[Sm(\textit{d}\text{-fbc})_2(L)(CF_3COO)]\cdot H_2O$		Complex 4 $[Eu(\textit{d}\text{-fbc})_2(L)(CF_3COO)]\cdot H_2O$	
**Bond**	**lengths (Å)**	**Bond**	**lengths (Å)**
Sm1−O1	2.460(6)	Eu1−O1	2.448(4)
Sm1−O2	2.471(6)	Eu1−O2	2.461(4)
Sm1−O3	2.375(6)	Eu1−O3	2.358(4)
Sm1−O4	2.461(6)	Eu1−O4	2.444(4)
Sm1−O5	2.393(6)	Eu1−O5	2.386(4)
Sm1−O6	2.360(6)	Eu1−O6	2.344(4)
Sm1−N1	2.645(8)	Eu1−N1	2.609(5)
Sm1−N2	2.770(9)	Eu1−N2	2.772(6)
Sm1−N3	2.621(8)	Eu1−N3	2.626(6)
Sm2−O9	2.443(7)	Eu2−O9	2.446(5)
Sm2−O10	2.362(7)	Eu2−O10	2.364(5)
Sm2−O11	2.462(7)	Eu2−O11	2.430(4)
Sm2−O12	2.398(7)	Eu2−O12	2.398(4)
Sm2−O13	2.427(7)	Eu2−O13	2.414(5)
Sm2−O14	2.343(7)	Eu2−O14	2.346(5)
Sm2−N5	2.612(8)	Eu2−N5	2.625(5)
Sm2−N6	2.767(11)	Eu2−N6	2.751(7)
Sm2−N7	2.627(8)	Eu2−N7	2.589(6)

* * O2, O3 and O4, O5 are from the two *d*-fbc, O6 is from trifluoroacetate, O1 and N2 are from benzohydazide and N1, N3 are from pyridines of ligand L.

.

Table S3. Selected angles (deg) of complexes 1 and 2.

Complex 1 $[La(d\text{-fbc})_2(L)(CF_3COO)]\cdot H_2O$ Angles(Å)				Complex 2 $[La(l\text{-fbc})_2(L)(CF_3COO)]\cdot H_2O$ Angles(Å)			
O2−La1−N2	111.78(10)	N7−La2−N6	61.08(11)	O1−La1−N3	71.8(2)	O11−La2−N6	112.5(2)
O2−La1−N1	134.26(10)	O9−La2−N7	71.78(10)	O1−La1−N2	60.0(2)	O11−La2−N5	134.4(2)
O2−La1−N3	72.79(10)	O9−La2−N6	60.11(10)	O1−La1−N1	82.1(3)	O11−La2−N7	72.9(2)
O6−La1−O2	74.62(10)	O9−La2−N5	82.42(11)	O4−La1−N3	68.1(3)	O14−La2−O11	74.4(2)
O6−La1−O3	95.23(11)	O13−La2−N7	68.50(11)	O4−La1−O1	131.4(2)	O14−La2−O10	95.5(3)
O6−La1−N2	135.02(10)	O13−La2−O9	131.61(10)	O4−La1−O2	126.9(3)	O14−La2−N6	135.3(2)
O6−La1−O4	76.14(11)	O13−La2−O11	126.85(11)	O4−La1−N2	117.6(3)	O14−La2−O13	75.9(2)
O6−La1−O1	141.78(11)	O13−La2−N6	118.19(11)	O4−La1−N1	143.3(2)	O14−La2−O9	142.0(2)
O6−La1−O5	81.55(11)	O13−La2−N5	143.12(10)	O5−La1−N3	81.8(2)	O14−La2−O12	81.0(3)
O6−La1−N1	144.80(11)	O12−La2−N7	82.08(10)	O5−La1−O1	129.0(3)	O14−La2−N5	144.7(3)
O6−La1−N3	81.16(11)	O12−La2−O9	129.34(12)	O5−La1−O4	70.5(2)	O14−La2−N7	81.8(3)
O3−La1−O2	69.99(9)	O12−La2−O13	70.61(11)	O5−La1−O2	138.9(2)	O10−La2−O11	69.7(2)
O3−La1−N2	129.38(10)	O12−La2−O11	139.01(10)	O5−La1−N2	69.0(3)	O10−La2−N6	128.9(2)
O3−La1−O4	70.19(9)	O12−La2−N6	69.32(11)	O5−La1−N1	76.1(3)	O10−La2−O13	70.5(2)
O3−La1−O1	77.54(10)	O12−La2−N5	76.17(11)	O3−La1−N3	81.5(3)	O10−La2−O9	77.4(2)
O3−La1−O5	139.98(10)	O10−La2−N7	81.32(11)	O3−La1−O1	75.8(2)	O10−La2−O12	140.6(2)
O3−La1−N1	81.36(11)	O10−La2−O9	75.75(11)	O3−La1−O4	72.3(2)	O10−La2−N5	81.3(3)
O3−La1−N3	142.18(10)	O10−La2−O13	72.01(10)	O3−La1−O5	142.7(2)	O10−La2−N7	141.8(2)
O4−La1−O2	127.31(10)	O10−La2−O12	142.46(10)	O3−La1−O2	69.8(2)	O13−La2−O11	126.8(3)
O4−La1−N2	119.94(10)	O10−La2−O11	69.96(9)	O3−La1−N2	128.2(2)	O13−La2−N6	119.8(2)
O4−La1−O1	132.66(9)	O10−La2−N6	128.23(10)	O3−La1−N1	140.3(2)	O13−La2−O9	132.9(2)
O4−La1−N1	69.73(11)	O10−La2−N5	140.41(10)	O2−La1−N3	137.6(2)	O13−La2−N5	69.9(3)
O4−La1−N3	142.45(9)	O11−La2−N7	137.19(10)	O2−La1−O1	71.3(2)	O13−La2−N7	142.9(2)
O1−La1−O2	67.60(10)	O11−La2−O9	70.83(11)	O2−La1−N2	114.9(3)	O9−La2−O11	68.1(2)
O1−La1−N2	59.70(10)	O11−La2−N6	114.34(11)	O2−La1−N1	72.0(2)	O9−La2−N6	59.5(2)
O1−La1−N1	71.98(10)	O11−La2−N5	71.83(11)	O6−La1−N3	143.0(3)	O9−La2−N5	71.9(2)
O1−La1−N3	82.41(10)	O14−La2−N7	143.48(11)	O6−La1−O1	143.6(3)	O9−La2−N7	81.7(2)
O5−La1−O2	143.89(10)	O14−La2−O9	143.34(11)	O6−La1−O4	76.0(3)	O12−La2−O11	143.3(2)
O5−La1−N2	68.10(10)	O14−La2−O13	76.13(11)	O6−La1−O5	78.2(3)	O12−La2−N6	68.3(2)
O5−La1−O4	70.34(10)	O14−La2−O12	77.82(12)	O6−La1−O3	96.0(3)	O12−La2−O13	70.6(2)
O5−La1−O1	127.50(11)	O14−La2−O10	96.54(12)	O6−La1−O2	72.6(3)	O12−La2−O9	127.6(3)
O5−La1−N1	79.21(11)	O14−La2−O11	72.86(11)	O6−La1−N2	135.6(3)	O12−La2−N5	79.9(2)
O5−La1−N3	77.09(10)	O14−La2−N6	134.97(11)	O6−La1−N1	82.6(3)	O12−La2−N7	77.0(2)
N1−La1−N2	61.52(11)	O14−La2−N5	81.71(12)	N1−La1−N3	122.2(3)	N5−La2−N6	61.2(3)
N3−La1−N2	60.72(10)	N5−La2−N7	122.49(13)	O1−La1−N3	61.4(3)	N7−La2−N6	60.6(2)
N3−La1−N1	122.15(12)	N5−La2−N6	61.46(11)	O1−La1−N2	71.8(2)	N7−La2−N5	121.8(3)

Table S4. Selected angles (deg) of complexes 3 and 4

**Complex 3** [Sm(*d*-fbc)$_2$(L)(CF$_3$COO)]·H$_2$O Angles(Å)				**Complex 4** [Eu(*d*-fbc)$_2$(L)(CF$_3$COO)]·H$_2$O Angles(Å)			
O6−Sm1−O3	95.0(3)	N7−Sm2−N6	62.4(3)	O1−Eu1−N2	61.46(15)	N5−Eu2−N6	62.72(18)
O6−Sm1−O2	73.9(2)	O9−Sm2−N7	72.7(2)	O1−Eu1−N3	72.15(16)	O10−Eu2−N5	79.73(17)
O6−Sm1−O5	80.6(3)	O9−Sm2−N6	62.1(2)	O1−Eu1−N1	85.13(15)	O10−Eu2−O13	70.26(15)
O6−Sm1−O1	141.6(2)	O9−Sm2−O11	70.4(2)	O6−Eu1−O1	141.21(16)	O10−Eu2−O11	71.93(15)
O6−Sm1−O4	75.1(2)	O9−Sm2−N5	84.8(3)	O6−Eu1−O2	73.74(16)	O10−Eu2−O9	74.50(17)
O6−Sm1−N3	80.1(2)	O13−Sm2−N7	68.8(3)	O6−Eu1−N2	135.32(16)	O10−Eu2−O12	142.57(17)
O6−Sm1−N2	135.3(2)	O13−Sm2−N6	119.7(3)	O6−Eu1−O5	80.69(17)	O10−Eu2−N7	141.36(16)
O6−Sm1−N1	144.3(3)	O13−Sm2−O9	131.2(2)	O6−Eu1−O4	75.35(16)	O10−Eu2−N6	128.56(16)
O3−Sm1−O2	71.49(19)	O13−Sm2−O11	125.7(3)	O6−Eu1−O3	94.68(17)	O13−Eu2−N5	68.59(18)
O3−Sm1−O5	141.1(2)	O13−Sm2−N5	142.4(2)	O6−Eu1−N3	144.15(17)	O13−Eu2−O11	125.97(19)
O3−Sm1−O1	76.4(2)	O11−Sm2−N7	138.1(2)	O6−Eu1−N1	79.71(16)	O13−Eu2−O9	131.03(15)
O3−Sm1−O4	69.0(2)	O11−Sm2−N6	114.1(3)	O2−Eu1−N2	111.50(16)	O13−Eu2−N7	142.01(16)
O3−Sm1−N3	142.4(2)	O11−Sm2−N5	70.4(2)	O2−Eu1−N3	134.75(15)	O13−Eu2−N6	119.90(17)
O3−Sm1−N2	129.5(2)	O12−Sm2−N7	83.0(2)	O2−Eu1−N1	71.00(15)	O11−Eu2−N5	137.95(15)
O3−Sm1−N1	79.8(3)	O12−Sm2−N6	69.0(3)	O5−Eu1−O1	129.43(17)	O11−Eu2−O9	70.31(16)
O2−Sm1−N3	71.4(2)	O12−Sm2−O9	131.0(3)	O5−Eu1−O2	140.67(15)	O11−Eu2−N7	70.28(17)
O2−Sm1−N2	111.8(2)	O12−Sm2−O13	72.2(3)	O5−Eu1−N2	68.17(16)	O11−Eu2−N6	113.70(17)
O2−Sm1−N1	134.7(2)	O12−Sm2−O11	137.2(2)	O5−Eu1−O4	72.20(16)	O14−Eu2−N5	142.63(18)
O5−Sm1−O2	140.7(2)	O12−Sm2−N5	75.2(3)	O5−Eu1−N3	82.01(17)	O14−Eu2−O10	95.67(19)
O5−Sm1−O1	129.0(3)	O10−Sm2−N7	80.3(2)	O5−Eu1−N1	75.45(16)	O14−Eu2−O13	74.93(17)
O5−Sm1−O4	72.5(2)	O10−Sm2−N6	129.0(2)	O4−Eu1−O1	131.75(14)	O14−Eu2−O11	71.97(17)
O5−Sm1−N3	75.3(2)	O10−Sm2−O9	74.8(2)	O4−Eu1−O2	126.93(16)	O14−Eu2−O9	142.24(17)
O5−Sm1−N2	68.0(2)	O10−Sm2−O13	70.3(2)	O4−Eu1−N2	120.87(16)	O14−Eu2−O12	78.45(18)
O5−Sm1−N1	82.0(2)	O10−Sm2−O11	71.5(2)	O4−Eu1−N3	69.52(17)	O14−Eu2−N7	80.2(2)
O1−Sm1−O2	67.9(2)	O10−Sm2−O12	142.3(2)	O4−Eu1−N1	141.68(15)	O14−Eu2−N6	135.55(17)
O1−Sm1−O4	131.9(2)	O10−Sm2−N5	141.0(2)	O3−Eu1−O1	76.32(15)	O9−Eu2−N5	72.61(16)
O1−Sm1−N3	84.7(2)	N5−Sm2−N7	124.9(3)	O3−Eu1−O2	71.86(14)	O9−Eu2−N7	85.49(18)
O1−Sm1−N2	61.1(2)	N5−Sm2−N6	62.6(3)	O3−Eu1−N2	129.71(15)	O9−Eu2−N6	61.99(16)
O1−Sm1−N1	71.9(2)	O14−Sm2−N7	142.9(3)	O3−Eu1−O5	140.69(15)	O12−Eu2−N5	83.15(16)
O4−Sm1−O2	126.5(2)	O14−Sm2−N6	135.5(2)	O3−Eu1−O4	68.90(14)	O12−Eu2−O13	72.55(17)
O4−Sm1−N3	141.9(2)	O14−Sm2−O9	142.0(2)	O3−Eu1−N3	79.48(17)	O12−Eu2−O11	137.11(15)
O4−Sm1−N2	121.0(2)	O14−Sm2−O13	75.0(2)	O3−Eu1−N1	142.52(15)	O12−Eu2−O9	130.75(19)
O4−Sm1−N1	70.0(3)	O14−Sm2−O11	71.6(3)	N3−Eu1−N2	63.14(18)	O12−Eu2−N7	74.68(18)
N3−Sm1−N2	62.1(2)	O14−Sm2−O12	78.4(3)	N1−Eu1−N2	62.35(16)	O12−Eu2−N6	68.84(17)
N3−Sm1−N1	124.7(3)	O14−Sm2−O10	95.3(3)	N1−Eu1−N3	125.4(2)	N7−Eu2−N5	125.6(2)
N1−Sm1−N2	62.7(3)	O14−Sm2−N5	80.7(3)	O1−Eu1−N2	61.46(15)	N7−Eu2−N6	63.00(18)

Table S5. Hydrogen-bonding Geometry (Å and °)

Compounds	Donor–H···accepter	D–H	H···A	D···A	D–H···A
**1**	Ow–H···O(7)	0.87	1.88	2.746(5)	171.7
	N(4)–H···Ow ^{i}	0.95	2.33	2.917(11)	119.2
**2**	Ow–H···O(7)	0.87	1.92	2.767(12)	165.2
	N(4)–H···Ow ^{ii}	0.88	2.11	2.852(13)	141.4
**3**	Ow–H···O(7)	0.85	1.96	2.725(12)	149.0
	N(4)–H···Ow ^{iii}	0.88	2.17	2.906(13)	140.6
**4**	Ow–H···O(7)	0.87	1.91	2.731(8)	155.9
	N(4)–H···Ow ^{iv}	0.88	2.16	2.899(9)	140.9

Symmetry code: (i) 1/2-X,-1/2+Y,1-Z; (ii) -1/2-X,-1/2+Y,-2-Z; (iii) 1/2-X,-1/2+Y,1-Z; (iv) 3/2-X,-1/2+Y,1-Z

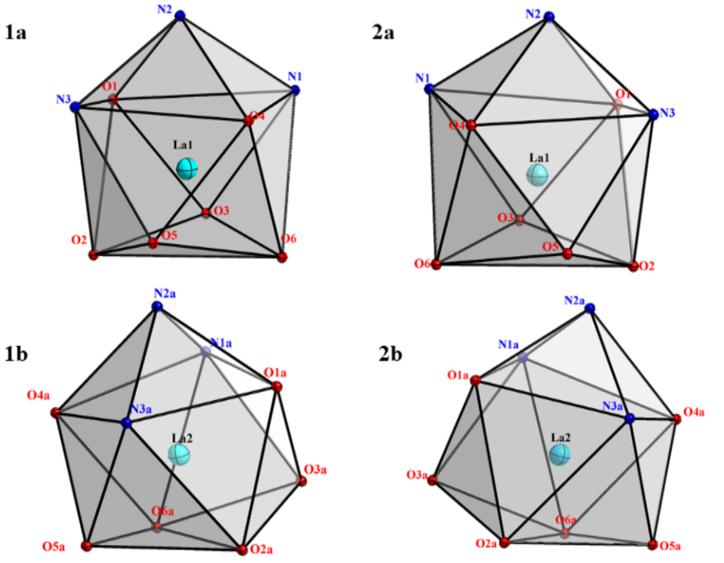

Figure S1. The polyhedron geometry of center La(III) of complex 1 (1a, 1b, left) and 2 (2a, 2b, right).

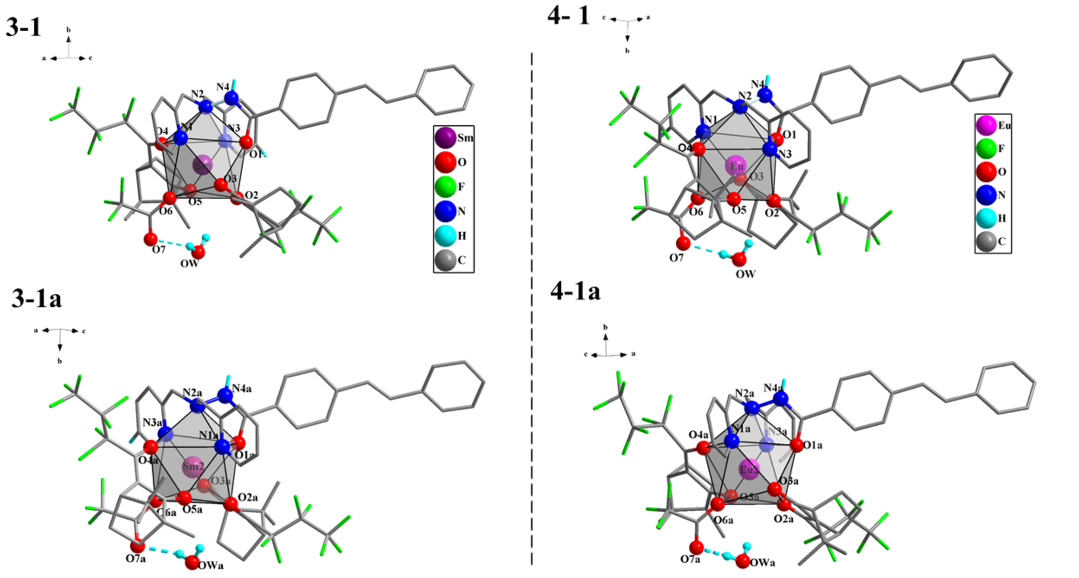

Figure S2. The Crystal structure and coordination polyhedron geometry of the two independent molecules of complexes 3 (3-1, 3-1a in left) and 4 (4-1, 4-1a in right) in the unit cell.

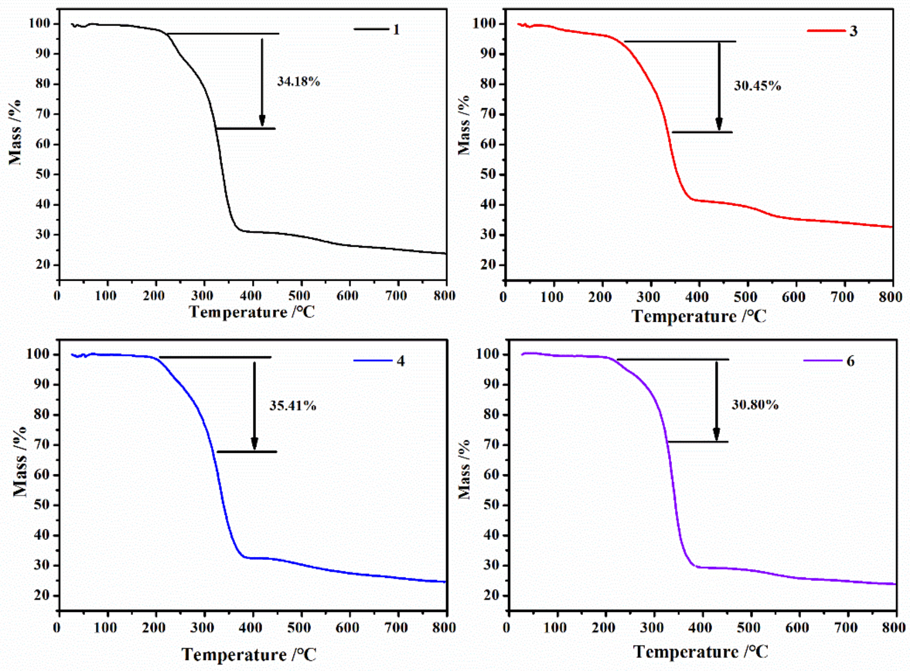

Figure S3. Thermogravimetric analysis (TGA) curves of complexes 1 (black), 3 (red), 4 (blue) and 5 (magenta).

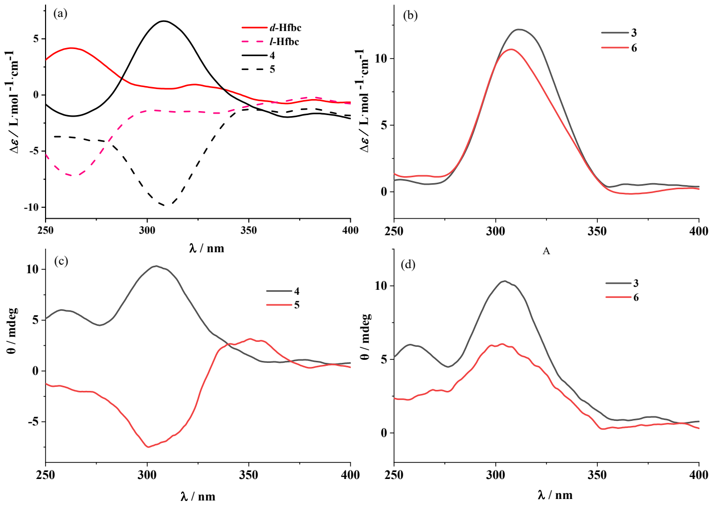

Figure S4. ECD spectra of *d/l*-Hfbc, complexes 4, 5 (a) and complexes 3, 6 (b) in acetonitrile solution, and complexes 4, 5 (c) and 3, 6 (d) in solid state, respectively.

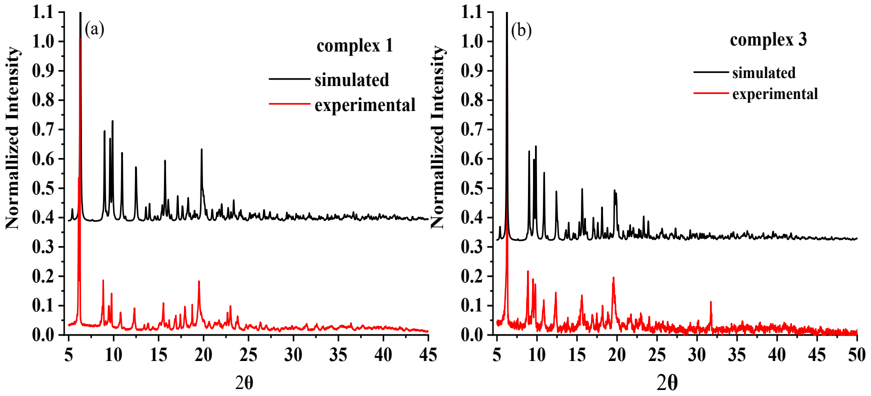

Figure S5. Observed (Red) and calculated (Black) X-ray powder diffraction patterns of complexes 1 (a) and 3 (b).

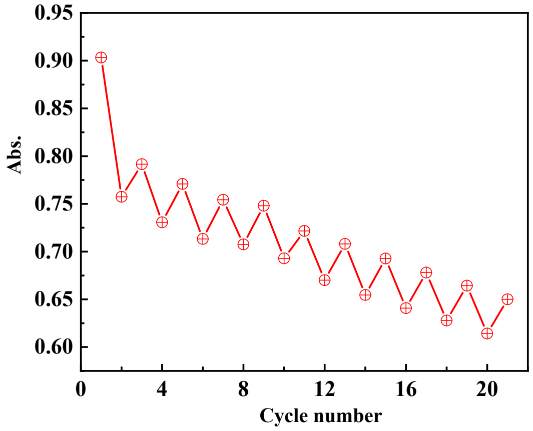

Figure S6. Cycles of maximum absorption intensity at 319 nm of ligand L in acetonitrile solutions are modulated by the irradiation of UV light at 365 nm for 10 min and 254 nm for 5 min, alternatively.

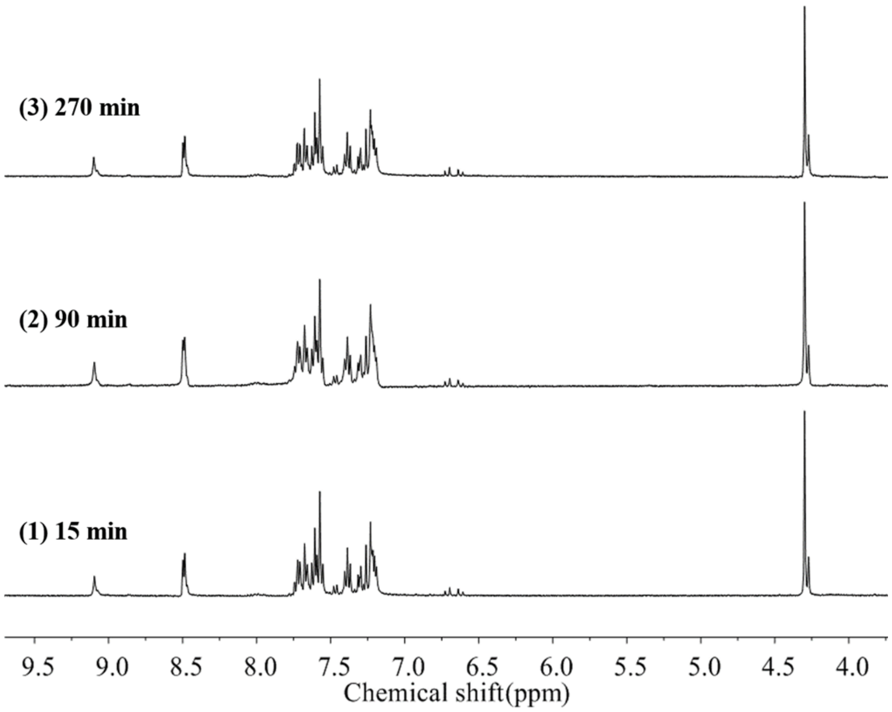

Figure S7. Evolution of ^{1}H-NMR spectra of L in acetonitrile-d_3 solution (after 365 nm light irradiation for 2 h) at 254 nm light with increasing irradiation time.

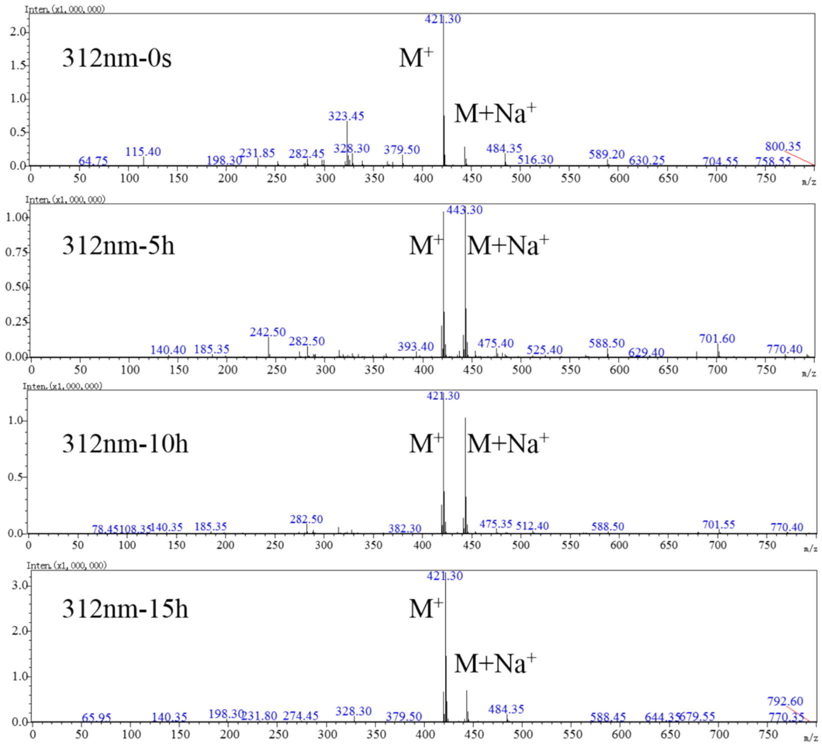

Figure S8. Mass spectra of L in methanol solution exposed to 312 nm UV light in different irradiation time.

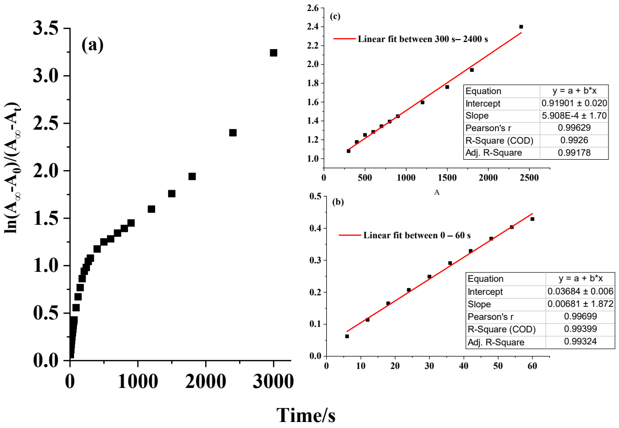

Figure S9. The *trans* to *cis* photoisomerization and photocyclization reactional kinetics of $\ln(A_{\infty}-A_0)/(A_{\infty}-A_t)$ as a function of time for L in acetonitrile solution.

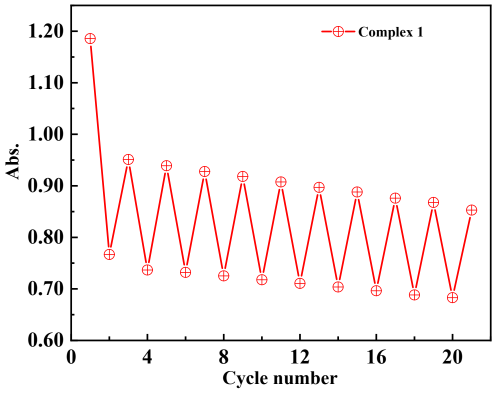

Figure S10. Cycles of maximum absorption intensity at 324 nm of complex 1 in acetonitrile solution are modulated by the irradiation of UV light at 365 nm for 3 min and 254 nm for 5 min, alternatively.

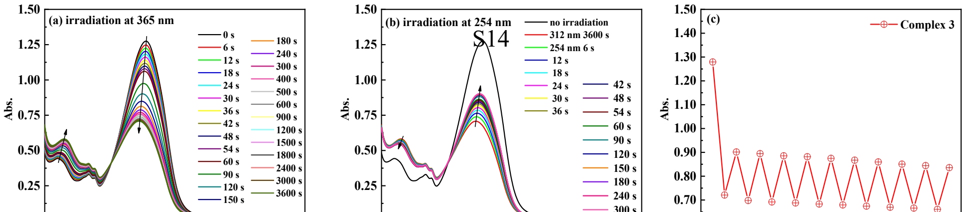

Figure S11. UV-Vis spectral change of complexes 3 (a, b), 4 (e, f), 6 (h, j) in acetonitrile solutions (2.0×10^{-5} mol·L^{-1}) upon irradiation at UV-365 nm and recoverable irradiation at UV-254 nm as a function of time and cycles of maximum absorption intensity at 324 nm are modulated by the irradiation of UV light at 365 nm for 3 min and 254 nm for 5 min, alternatively (c for complex 3, g for complex 4, k for complex 6).

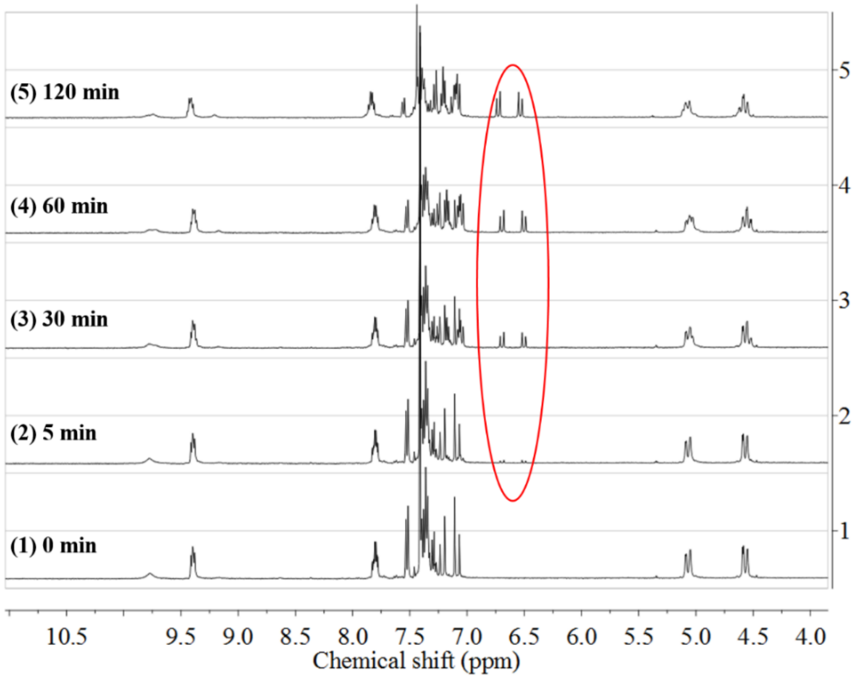

Figure S12. Evolution of ^{1}H-NMR spectra of complex 1 in acetonitrile-d_3 solution at 365 nm light with increasing irradiation time.

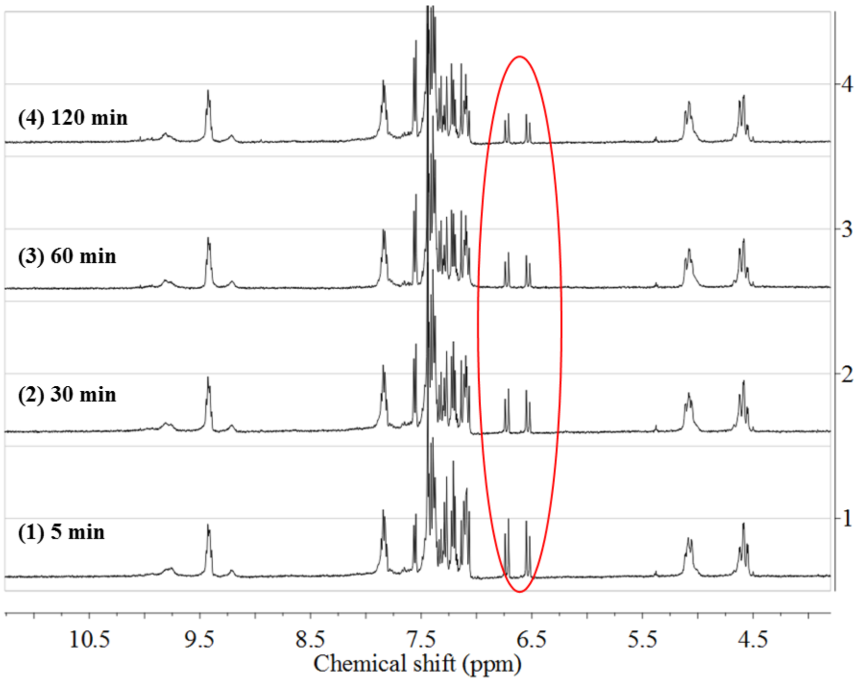

Figure S13. Evolution of ^{1}H-NMR spectra of complex 1 in acetonitrile-d_3 solution (after 365 nm light irradiation for 2 h) at 254 nm light with increasing irradiation time.

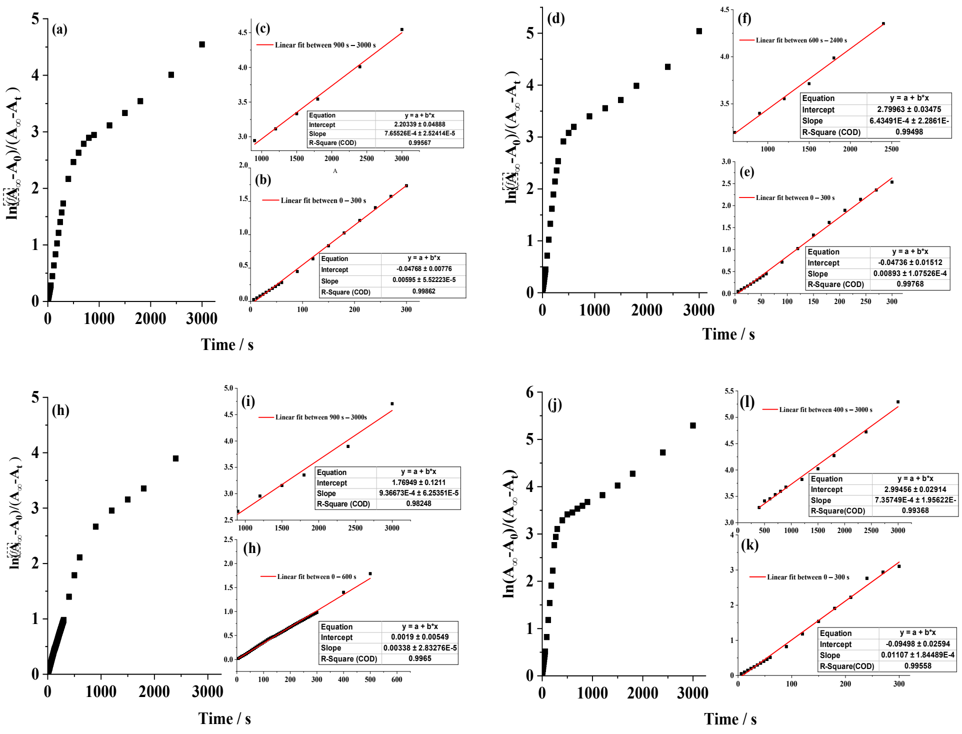

Figure S14.The *trans* to *cis* photoisomerization kinetics of $\ln(A_\infty - A_0)/(A_\infty - A_t)$ as a function of time for complexes 1 (a, b, c), 3 (d, e, f), 4 (g, h, i), and 6 (j, k, l) in acetonitrile solutions.

Table S6. The main structural geometric parameters obtained by the RB3LYP functional method for the optimization of complex 3 and the crystallographic data measured by single crystal X-ray diffractometer.

Bond	Bond lengths (Å)		Bond	Bond angle (°)	
	measured	RB3LYP		measured	RB3LYP
$Sm_1–O_1$	2.460	2.495	$O_2–Sm_1–N_2$	111.8	118.6
$Sm_1–O_2$	2.471	2.484	$O_6–Sm_1–O_2$	73.9	71.6
$Sm_1–O_3$	2.375	2.353	$O_6–Sm_1–O_3$	95.0	105.8
$Sm_1–O_4$	2.461	2.460	$O_6–Sm_1–O_4$	75.1	76.9
$Sm_1–O_5$	2.393	2.392	$O_6–Sm_1–O_5$	80.6	77.1
$Sm_1–O_6$	2.360	2.348	$O_6–Sm_1–N_2$	135.3	128.7
$Sm_1–N_1$	2.645	2.724	$O_2–Sm_1–N_1$	71.4	73.8
$Sm_1–N_2$	2.770	2.870	$O_3–Sm_1–N_2$	129.5	125.3
$Sm_1–N_3$	2.621	2.728	$O_6–Sm_1–N_3$	144.3	147.8
Trans C=C	1.310	1.351	$N_1–Sm_1–N_3$	124.7	121.3

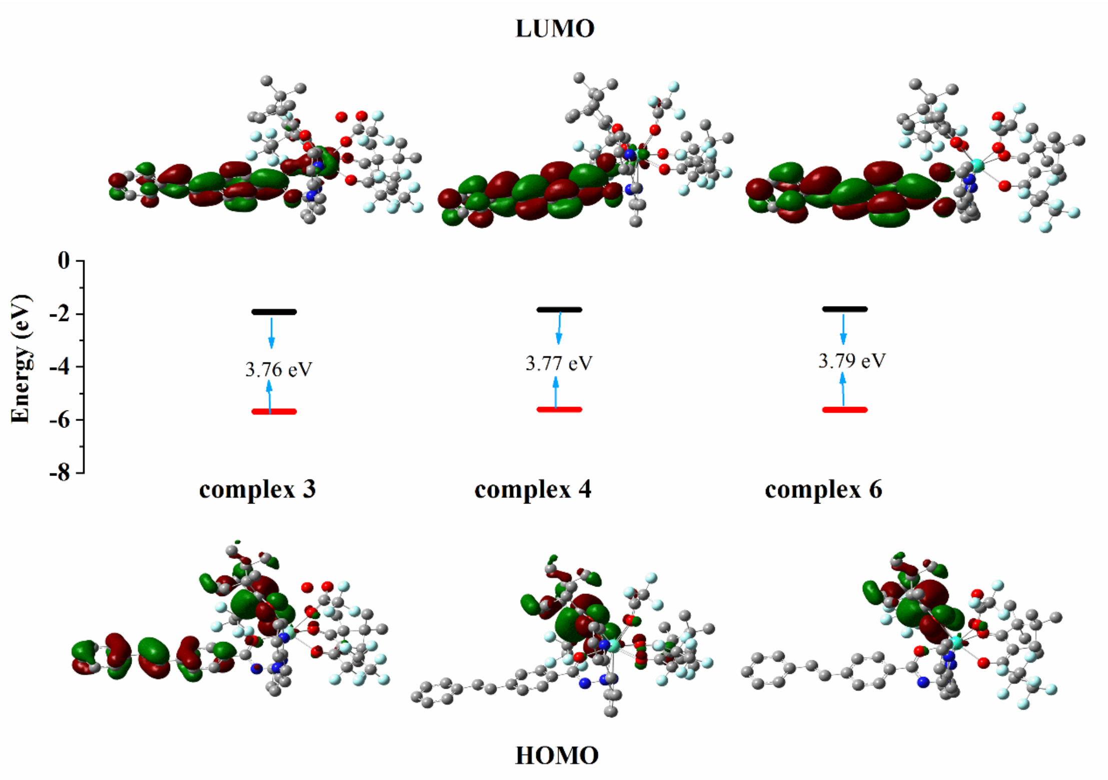

Figure S15. HOMO and LUMO energy levels and orbital distributions of complexes 3, 4 and 6.

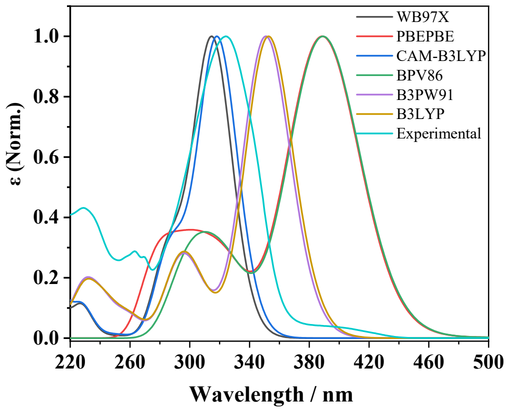

Figure S16. Overlay of calculated and experimental absorption spectra of the complex 1 in acetonitrile solutions.

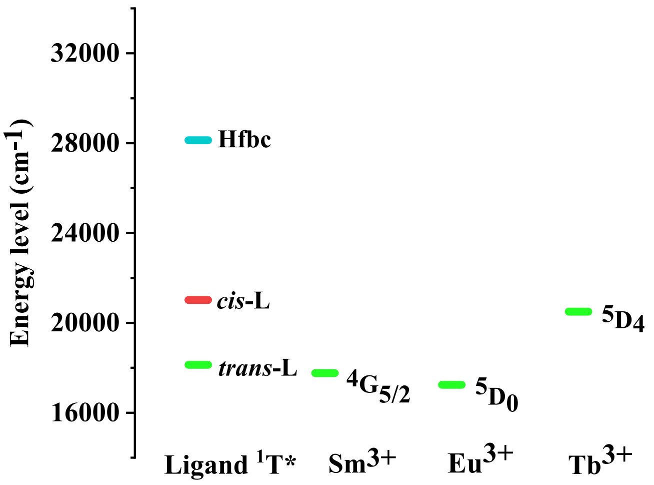

Figure S17. The energy levels of the first excited states of ligands *trans*-L, *cis*-L, Hfbc and the related energy levels of the Sm^{3+}, Eu^{3+} and Tb^{3+}.

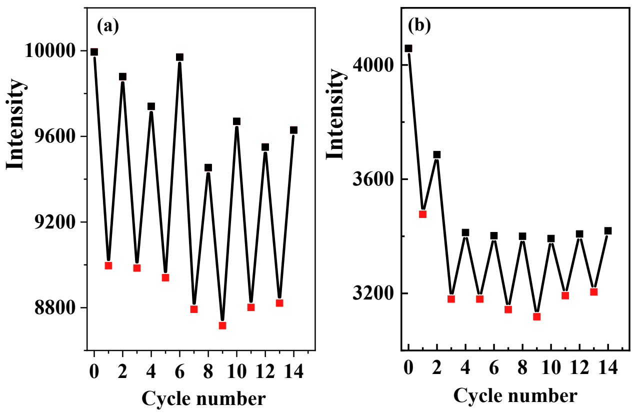

Figure S18. Cycles of maximum luminescence intensity at 420 nm are modulated by the irradiation of UV light at 365 nm for 2 min and 254 nm for 2 min, alternatively (a for complex 3, b for complex 4).
